# Evidence for autotrophic growth of purple sulfur bacteria using pyrite as electron and sulfur source

**DOI:** 10.1128/aem.00863-24

**Published:** 2024-06-20

**Authors:** Hugo V. Alarcon, Jonathon E. Mohl, Grace W. Chong, Ana Betancourt, Yi Wang, Weinan Leng, Jason C. White, Jie Xu

**Affiliations:** 1 Environmental Science and Engineering Program, the University of Texas at El Paso, El Paso, Texas, USA; 2 Department of Mathematical Sciences, the University of Texas at El Paso, El Paso, Texas, USA; 3 Border Biomedical Research Center, the University of Texas at El Paso, El Paso, Texas, USA; 4 Department of Earth, Environmental and Resource Sciences, the University of Texas at El Paso, El Paso, Texas, USA; 5 The Connecticut Agricultural Experiment Station, New Haven, Connecticut, USA; 6 The National Center for Earth and Environmental Nanotechnology Infrastructure, Blacksburg, Virginia, USA; 7 School of Molecular Sciences, Arizona State University, Tempe, Arizona, USA; Colorado School of Mines, Golden, Colorado, USA

**Keywords:** purple sulfur bacteria, pyrite, anoxygenic photosynthesis, transcriptomic sequencing, cytochrome, iron sulfur cluster, *Allochromatium vinosum*

## Abstract

Purple sulfur bacteria (PSB) are capable of anoxygenic photosynthesis via oxidizing reduced sulfur compounds and are considered key drivers of the sulfur cycle in a range of anoxic environments. In this study, we show that *Allochromatium vinosum* (a PSB species) is capable of autotrophic growth using pyrite as the electron and sulfur source. Comparative growth profile, substrate characterization, and transcriptomic sequencing data provided valuable insight into the molecular mechanisms underlying the bacterial utilization of pyrite and autotrophic growth. Specifically, the pyrite-supported cell cultures (“py”’) demonstrated robust but much slower growth rates and distinct patterns from their sodium sulfide-amended positive controls. Up to ~200-fold upregulation of genes encoding various *c*- and *b*-type cytochromes was observed in “py,” pointing to the high relevance of these molecules in scavenging and relaying electrons from pyrite to cytoplasmic metabolisms. Conversely, extensive downregulation of genes related to LH and RC complex components indicates that the electron source may have direct control over the bacterial cells’ photosynthetic activity. In terms of sulfur metabolism, genes encoding periplasmic or membrane-bound proteins (e.g., FccAB and SoxYZ) were largely upregulated, whereas those encoding cytoplasmic proteins (e.g., Dsr and Apr groups) are extensively suppressed. Other notable differentially expressed genes are related to flagella/fimbriae/pilin(+), metal efflux(+), ferrienterochelin(−), and [NiFe] hydrogenases(+). Characterization of the biologically reacted pyrite indicates the presence of polymeric sulfur. These results have, for the first time, put the interplay of PSB and transition metal sulfide chemistry under the spotlight, with the potential to advance multiple fields, including metal and sulfur biogeochemistry, bacterial extracellular electron transfer, and artificial photosynthesis.

**IMPORTANCE:**

Microbial utilization of solid-phase substrates constitutes a critical area of focus in environmental microbiology, offering valuable insights into microbial metabolic processes and adaptability. Recent advancements in this field have profoundly deepened our knowledge of microbial physiology pertinent to these scenarios and spurred innovations in biosynthesis and energy production. Furthermore, research into interactions between microbes and solid-phase substrates has directly linked microbial activities to the surrounding mineralogical environments, thereby enhancing our understanding of the relevant biogeochemical cycles. Our study represents a significant step forward in this field by demonstrating, for the first time, the autotrophic growth of purple sulfur bacteria using insoluble pyrite (FeS2) as both the electron and sulfur source. The presented comparative growth profiles, substrate characterizations, and transcriptomic sequencing data shed light on the relationships between electron donor types, photosynthetic reaction center activities, and potential extracellular electron transfer in these organisms capable of anoxygenic photosynthesis. Furthermore, the findings of our study may provide new insights into early-Earth biogeochemical evolutions, offering valuable constraints for understanding the environmental conditions and microbial processes that shaped our planet’s history.

## INTRODUCTION

Purple bacteria are photosynthetic, Gram-negative prokaryotes that convert light energy into chemical energy through the process of anoxygenic photosynthesis (1). Anoxic conditions are required for purple bacteria to grow phototrophically, as the biosynthesis of their pigments and complexes is repressed by molecular oxygen (2). Although purple bacteria can utilize a wide range of electron donors to couple their autotrophic CO_2_ fixation, a subgroup preferentially oxidizes reduced sulfur compounds (i.e., hydrogen sulfide) during their phototrophic growth and are named purple sulfur bacteria (PSB). Almost all identified PSB belong to *Chromatiaceae*, *Ectothiorhodospiraceae,* or *Halorhodospiraceae* families (3). A key difference between these families of PSB lies in the location of the sulfur globules formed during the bacterial growth on reduced sulfur (4), which occur intracellularly in members of *Chromatiaceae* but extracellularly in those of *Ectothiorhodospiraceae/ Halorhodospiraceae*. The specific strain studied in this reported work, *Allochromatium vinosum* DSM180, belongs to *Chromatiaceae*. Purple sulfur bacteria can thrive in various freshwater, marine, and hypersaline environments that contain hydrogen sulfide and are illuminated, usually inhabiting the stratum below oxygenic phototrophs. A consequence of this is that the wavelengths of light reaching purple sulfur (and non-sulfur) bacteria are limited, due to the absorption of the blue and red regions in the solar spectrum by the chlorophyll-containing oxygenic phototrophs (5). The most essential pigments in PSB are capable of absorbing near-infrared and green light and use it for anoxygenic photosynthesis. PSB are key participants in the anoxic cycling of carbon, mostly as primary producers fixing CO_2_ and occasionally as light-stimulated consumers of reduced organic compounds (6–9). The most critical roles of PSB in ecosystems, however, lie in their capability of reoxidizing hydrogen sulfide produced by sulfate-reducers (1). Hydrogen sulfide is a highly poisonous substance for most biota. The reoxidation of sulfide by PSB yields nontoxic forms of sulfur, such as elemental sulfur (S^0^) and sulfate (SO_4_^2−^), thereby detoxifying the associated environments and importantly closing the essential sulfur oxidation-reduction cycle.

Photosynthetic pathways in phototrophic purple bacteria (including PSB) have been studied for decades (10–17). Here, we will briefly describe the phototrophic pathway in PSB. In PSB, incident photons are absorbed by an array of light-harvesting (LH) complexes within the intracytoplasmic membrane. These complexes consist of proteins that contain bacteriochlorophyll (BChl) and carotenoid pigments, which can absorb light energy by transforming their bonding and electronic states and funneling it down an energy gradient to a central reaction center (RC). In RC, charge separation occurs across the membrane and drives a series of redox reactions involving other biomolecules or protein complexes such as quinone/quinol, cytochrome *b/c*, and cytochrome *c* complexes bound within the membrane. Along with electron transport, proton motive force (PMF) is formed and powers ATP synthase complexes. Weissgerber et al. (18) sequenced and annotated the full genome of *A. vinosum,* identifying three subunits of the RC, *pufC*, *pufM,* and *pufL,* which are clustered and co-transcribed with three sets of *pufA* and *pufB* genes encoding light-harvesting complex (LH1) apoproteins (19). Six potential *puc* gene pairs were also identified that encode α- and β-apoproteins for several LH2 complex types (20). It was reported that *A. vinosum* produces one type of bacteriochlorophyll, namely BChl*a*, and carotenoids of the spirilloxanthin series (18).

A central feature of PSB is their capability to oxidize reduced sulfur compounds during photolithoautotrophic growth. The known substrates that can be used by PSB include sulfide, polysulfides, elemental sulfur, and thiosulfate (21). In terms of sulfide oxidation, *A. vinosum* has the genetic capacity to form several different enzymes, including the periplasmic flavocytochrome *c* sulfide dehydrogenase (Fcc), and membrane-bound sulfide:quinone-oxidoreductases (Sqr), which are predicted to be oriented toward the periplasm (22, 23). *A. vinosum* was also shown to contain the genetic information for rhodaneses, sulfur relay proteins, and polysulfide reductase-like proteins with unknown functions (24–27). Some PSB including *A. vinosum* have been shown to oxidize externally supplied elemental sulfur (28). However, controversy exists regarding whether PSB may utilize commercially available elemental sulfur, and it remains unknown how PSB may bind, activate, and take up solid-phase sulfur. In principle, bacterial cells may interact with their insoluble substrate through direct physical contact via outer membrane proteins or through excreting extracellular substances that solubilize the substrate. For *A. vinosum*, evidence for the formation of soluble intermediates like sulfide or polysulfides during uptake of elemental sulfur was not obtained (29), rendering direct cell-sulfur contact as a likely option for the cells’ interaction with the solid substrate. It was also shown that *A. vinosum* strongly prefers the polymeric sulfur fraction (i.e., sulfur chains) of the elemental sulfur and is likely unable to utilize the S_8_ rings (30). Regarding sulfur-oxidation in *A. vinosum*, many of the former studies have also focused on the mechanisms involved in their sulfur globule utilization and proposed that the dissimilatory sulfite reductase (Dsr) system might play essential roles as several *dsr*-deleted mutants of *A. vinosum* were found unable to degrade these globules (31–34).

**Fig 1:**
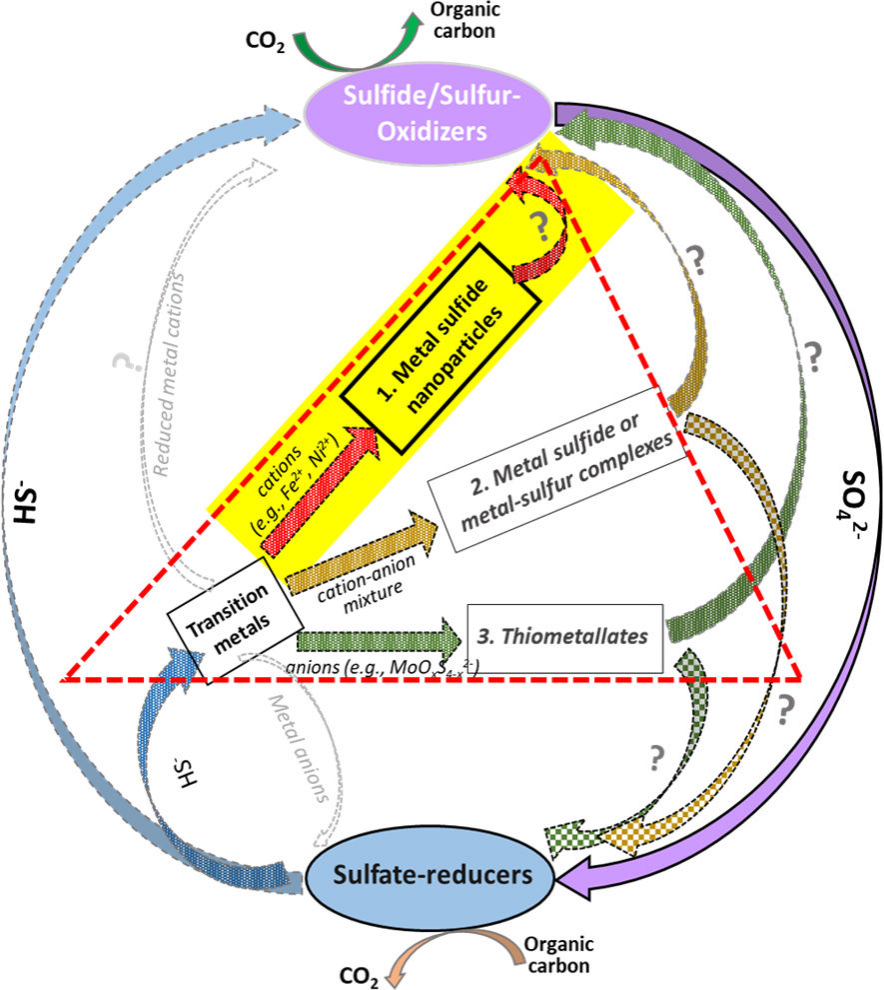
Microbial sulfur oxidation-reduction patterns complicated by the presence of transition metal species (TMs). In the absence of TMs, sulfate reducers reduce sulfate to sulfide/elemental sulfur in couple with heterotrophy or mixotrophy, whereas sulfur-oxidizers oxidize sulfide/elemental sulfur back to sulfate in couple with autotrophy. In the presence of TMs, TM sulfide nanoparticles or thiometallate clusters may form within the cycle. It is unknown how the formed TM-sulfur nanoparticles or complexes may affect the metabolic activity of associated sulfur-oxidizers that depend on “free” sulfide to support CO_2_ fixation.

It remains unknown if *A. vinosum* or other PSB are capable of utilizing other solid-phase substrates besides elemental sulfur. In the various habitats of PSB through geological time, there had been, and still are, high chances of metal sulfide formation, which may divert free sulfide out of the sulfur cycle and complicate the associated metal-sulfur geochemistry (Fig. 1). Interaction of PSB with metal sulfides in general, therefore, may have its evolutionary basis, especially considering the prevalence and transformations of sulfide-dominated environments on early Earth. Based on previous studies (35, 36), the oceans during the Mesoproterozoic Era were overall constrained to support a mix of sulfidic, ferruginous, and oxic conditions. Later statistical treatment of the available iron speciation data suggests that euxinic conditions were relatively common (37, 38), which may have provided a strong sink for Fe(II), leading to extensive FeS formation. For modern geochemical settings, partial documentation of coexistence of Fe sulfide precipitates and microbial sulfide oxidation (including phototrophic) was available for euxinic or ferruginous lakes (39–41), fjords (42–44), estuaries (45–47), and shallow marine basins (48–50). Iron monosulfide in geochemical setting is a metastable phase and will eventually transform into greigite and pyrite (51–55).

Here, we present the first evidence for *A. vinosum*’s capability of utilizing solid-phase metal sulfide, that is, pyrite (FeS_2_) and provide thorough transcriptomic profiling and substrate characterization data. We confirmed robust but much slower growth of the pyrite-supported cell cultures (“py”) compared with their positive controls (amended with sodium sulfide and containing soluble ∑H_2_S). Differential gene expression analyses (of cells harvested at their respective exponential growth phases in “py” versus positive controls) revealed up to hundreds of fold changes in the expression of genes encoding various types of cytochromes, LH complex subunits, bacteriochlorophyll *a*, and enzymes involved in dissimilatory sulfur metabolism. We have also proposed a model for pyrite oxidation by *A. vinosum* in the discussion.

## MATERIALS AND METHODS

### Strain, medium, and culture conditions

The strain of *A. vinosum* DSM 180 was obtained from the Leibniz Institute DSMZ, Germany. Culture media for *A. vinosum* was prepared following Pfenning’s medium recipe with modifications that removed compounds allowing for potential heterotrophic growth. Several types of media were prepared for the experiments: one for the sulfur-free control, another for the positive controls, and the last for the pyrite-amended cell cultures. Other than the sulfur source, these media are identical in their compositions. Specifically, the positive control medium is amended with Na_2_S∙9H_2_O, overall consisting of 1.7 mM of CaCl_2_∙2H_2_O, 250 mg/L of yeast extract, 6.5 mM of NH_4_Cl, 4.6 mM of KCl, 1 mM of MgCl_2_∙6H_2_O, 20 mM of HEPES, 35 mM of NaHCO_3_, 5.1 mM of KH_2_PO_4_, and 5 mM of Na_2_S∙9H_2_O. The “py” medium did not contain Na_2_S∙9H_2_O but 750 mg/L of pyrite. The sulfur-free control contained neither Na_2_S∙9H_2_O nor pyrite. In preparation for the full media, we made two types of solutions (A and B) separately. Solution A was prepared through boiling Milli-Q water (18.2 MΩ∙cm), degassed with ultrapure N_2_ gas during cooling down. All salts except for the carbon and sulfur sources (i.e., NaHCO_3_ and Na_2_S∙9H_2_O/pyrite) and KH_2_PO_4_ were added to the degassed solution, which was further degassed using N_2_ for ~45 min. A mineral mix (composition provided in Supplemental Information) was added to the cooled solution A at a ratio of 10 µL/mL, following which trace amounts of concentrated 6N HCl were added (at a ratio of 1 µL/mL before bottling in serum bottles sealed by rubber septa and aluminum rings. The purpose of adding trace amounts of HCl is 2-fold: facilitating the dissolution of all the salts and resulting in a final medium (through mixing A and B) pH in the range of 7.1–7.3. As a separate solution (B), boiled and N_2_-degassed/cooled Milli-Q water was sterilized using 0.2 µm syringe filters and stored in a sterile serum bottle, further bubbled using ultrapure N_2_ at room temperature for ~15 min. Immediately prior to sealing the bottles with rubber septa, NaHCO_3_ and Na_2_S∙9H_2_O were added. The fast sealing can prevent the loss of sulfide and CO_2_, keeping the medium composition close to the designated one. Full media was made by mixing 90% of solution A into 10% of solution B by volume and adding 10 µL of ATCC Vitamin mix per mL of full media through syringe filtering. Inoculations of the positive and negative controls were carried out by adding 1% (vol/vol) of the stock cell culture medium (a positive control at mid-late exponential growth phase), and the initial total volume of cell cultures was 200 mL. Two types of negative controls were created: non-inoculated culture of pyrite-amended medium and inoculated culture of the sulfur-free medium (no sodium sulfide or metal sulfide added). The pyrite tested in the experiments was obtained from Fisher Scientific (as high purity naturally occurring pyrite FeS_2_, further pulverized and washed/sterilized using ethanol). X-ray diffraction and transmission electron microscopy coupled with energy dispersive spectroscopy analyses confirmed the pure pyrite (FeS_2_) phase. Inoculation of the pyrite-amended medium was done using a former pyrite cell culture (1%, vol/vol) to minimize potential sulfur carryover. The pyrite-to-pyrite inoculation was also observed, leading to slightly faster growth cycles of the pyrite cell cultures through multiple rounds of experiments. All “py” cell cultures and positive and negative controls were kept in a shaker incubator maintained at 30°C and 100 rpm, under an incandescent lamp with tungsten filament (100W, hyperspectral analysis of the light illumination is included in Supplemental Information). We note that the addition of a low amount of yeast extract (250 mg/L) is necessary to kick off the cell growth in the “py” samples. Cell growth was observed in the negative control but was significantly lower than that in “py.” The growth of the cell cultures was monitored using optical density at 600 nm and DNA yields (see the following section for DNA extraction and quantification). Bacteriochlorophyll a level of the cell cultures at various times was also evaluated using an acetone-methanol extraction method coupled with spectrophotometric analysis, but only in a comparative manner. It is noted that all the glassware used in the experiments is acid-washed and thoroughly rinsed with DI water and type-1 ultrapure water in our laboratory.

### Nucleic acid extraction and analysis

DNA and RNA samples were recovered from the cell cultures using the GenElute Bacterial Genomic DNA kit (Sigma Aldrich) and the RNeasy Mini kit (Qiagen), respectively. In sampling, 1 mL aliquots of the cell culture medium were removed using N_2_-purged syringes. In the case of sampling for RNA extraction, RNAProtect was immediately added to the aliquots and incubated for 5 min. The sampled aliquots (with/without RNAProtect) were then centrifuged at 5,000 g for 10 min, following which the supernatant was discarded. The cell pellets were maintained at −80°C until the DNA/RNA extraction was done. For the DNA extraction, the cell pellets were extracted using the Gram-positive quick protocol (which was found to be more efficient than the Gram-negative protocol for *A. vinosum*) from the GenElute Bacteria Genomic DNA kit manual. For the RNA extraction, the cell pellets were first lysed following a protocol recommended by the RNEasy kit. The lysis solution was prepared by mixing 10 µL of proteinase K (20 mg/mL) and 100 µL of lysozyme (15 mg/mL) in the TE buffer solution (10 mM of Tris-HCl, 1 mM of ethylenediaminetetraacetic acid, and pH 8). Enzymatic digestion was carried out at room temperature for 10 min in a rotary shaker. The RNeasy extraction was subsequently done using the lysate following the RNeasy Mini kit instructions. For the RNA extraction, DNA removal steps are included in the kit instructions. (We have tested multiple RNA extraction protocols, and the RNeasy was shown to be the most robust and consistent for *A. vinosum*.) Quantification of DNA and RNA, respectively, was done using Nanodrop One spectrophotometer and Qubit fluorometer, whereas quality control was done through 260/280 and 260/230 ratios and DIN and RIN analysis using Tapestation 2200.

### Transcriptomic sequencing and bioinformatics

**Fig 2:**
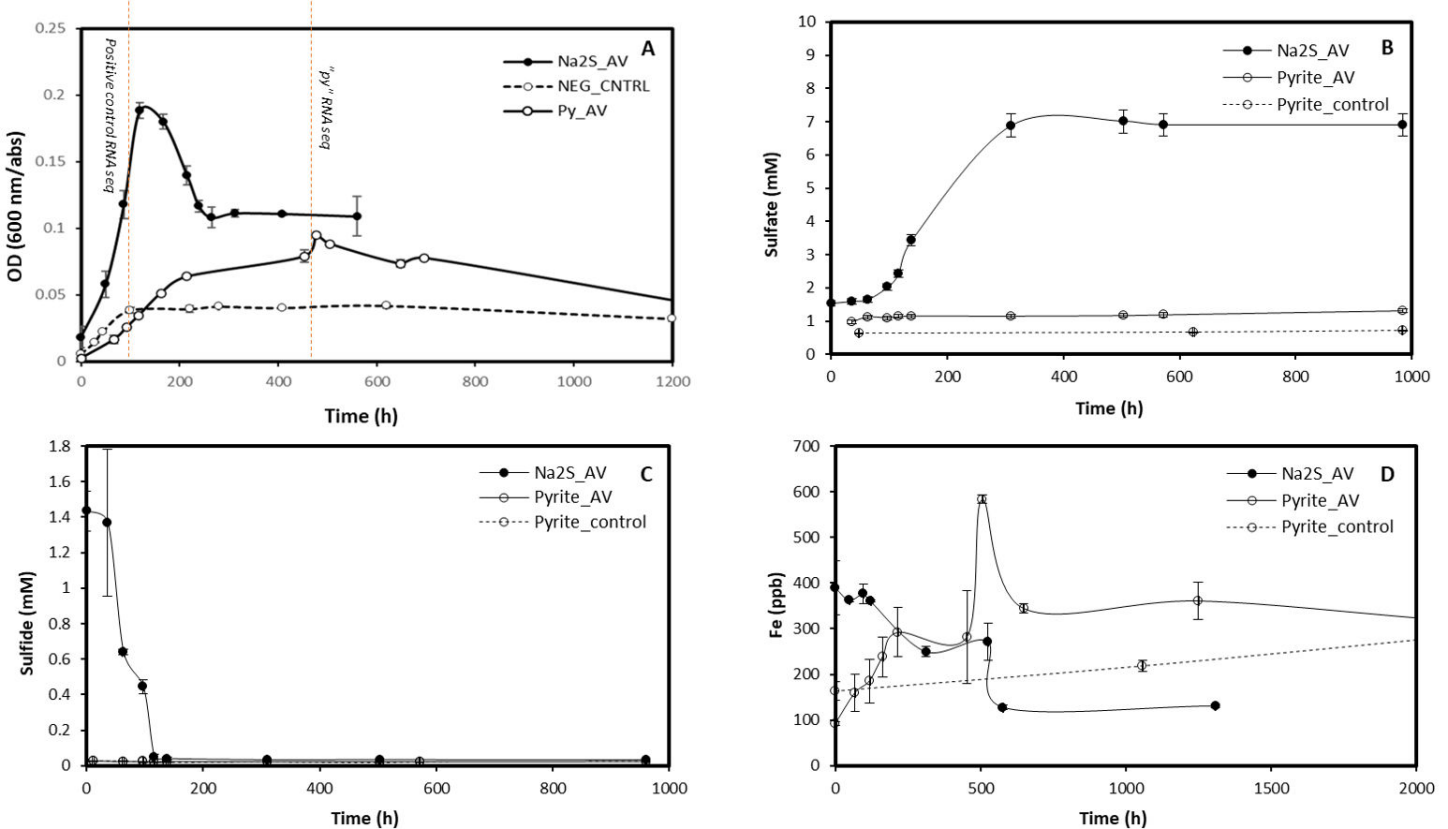
*A. vinosum* growth profiles. Time profiles of (**A**) optical density (600 nm) measurements of 10-fold diluted samples, (**B**) sulfate concentrations, (**C**) sulfide concentrations (2-fold dilution), and (**D**) iron concentrations. The red dashed lines in (**A**) mark the corresponding time points for the obtained transcriptomic sequencing data for “py“ and positive controls.

The Illumina platform technology was used to sequence both “py” samples and positive controls cDNA libraries, which were derived from total RNA cultures in their respective mid-late logarithmic phase (the time point for the samples used for transcriptomic sequencing are marked in Fig. 2). We have included triplicate samples for each sample type and triplicate sequencings for each sample. The sequencing was performed using a 400M read flow cell NextSeq 2000 cartridge with a 150 bp-end read length. To ensure the quality of the samples, FastQC was utilized to confirm their integrity. Trimmomatic software (56) was employed to trim reads of adapters, low-quality bases, and fragments smaller than 60 bp from raw data. The resulting high-quality trimmed reads were then aligned to the reference genome of *A. vinosum* (Genbank: CP001896.1) using Bowtie2 software (57), which can generate an indexed version of the reads. For transcript quantification, the paired-end indexed data of both positive control and pyrite samples were used with RNA-Seq by Expectation-Maximization (RSEM) software. Furthermore, the DESeq2 package (58) of the R was used for data normalization and differential analysis of the statistical processing of the data.

### Dissolved species characterization

Growth of bacteria in the positive controls and “py” samples was tracked indirectly by measuring the concentrations of dissolved iron, sulfide, and sulfate in the medium solution over time. Collected samples for sulfide measurements were processed immediately to minimize sulfide escape from solution over time. A 100 µL aliquot of each sample was reacted with 40 µL of excess zinc chloride solution (~ 100-fold of the molar amount of sulfide) to form metastable ZnS. Sulfide measurements were done using the methylene blue method, which involves a reagent to react with any sulfide present including the precipitated ZnS to yield the equimolar amount of methylene blue. The concentrations of the generated methylene blue were then measured by tracking the absorption at 665 nm using a MultiSkan UV-Vis spectrophotometer. Specifically, the zinc chloride-stabilized mixture was reacted with 250 µL of sulfide 1 reagent and 250 µL of sulfide two reagents (obtained from Hach) and diluted with 400 µL MQ water to generate a ~1:10 dilution. The mixture was placed in a rotary shaker for a period of 10 min before the UV-vis measurement. In the case of sulfate measurement, a nitrogen-purged syringe was used to collect aliquots of samples that were diluted 1:10 with Milli-Q water and subsequently filtered. Sulfate measurements were performed using a Dionex ICS-2100 ion chromatography system, and quality control (QC) was performed by jointly running a standard curve made with sodium sulfate. Concentrations of major elements in the control and sample solutions were measured using inductively coupled plasma (ICP)-optical emission spectroscopy (OES) or mass spectrometry (MS) depending on the concentration levels. The aliquots of the medium solution for the ICP runs were diluted 100-fold using 2% HNO_3_ solution and filtered (0.2 µM cutoff) into 15 mL conical centrifuge tubes. Samples were analyzed by ICP-OES (iCAP 6500, Thermo Fisher Scientific, Waltham, MA) and ICP-MS (7700 Series, Agilent, Santa Clara, CA) to determine the concentration levels for a group of elements (including Ca, K, Mg, Na, P, S, Zn, Fe, Ni, Mo, and Cu). To validate measurements, blank and standard reference materials (NIST-SRF 1570 and 1547, Metuchen, NJ) were prepared and analyzed. Spikes at different concentrations were used to obtain the standard working curve. The recovery rate of all the tested elements was above 99%. Yttrium (Y) was used as an internal standard, and a continuing calibration verification (CCV) sample was analyzed every 15 samples.

### Solid-phase characterization

Solid phases in the cell-free negative control and “py” samples were analyzed using X-ray diffractometry (XRD), transmission electron microscopy (TEM), and X-ray photoelectron spectroscopy (XPS). The solid pellets recovered through centrifugation and supernatant removal were processed using 0.1% Triton X-100 solution containing 10 µg/mL of lysozyme and 10 µg/mL of proteinase K to remove the bacterial cells and biomolecular debris. The pellets were sonicated in the processing solution for 45 min at room temperature. The solid particles were then separated by centrifuging the digestion mixture at 10,000 × *g* for 5 min at room temperature and removing the supernatant. The separate solid particles were washed twice with 0.1% Triton-X. All operations were carried out in an anaerobic chamber in sealed containers prepared to prevent sample oxidation. The biomass-digested solid particles were fractioned for XRD, XPS, and TEM analyses. The sample preparation for the XPS specimen involved drying the separated particles on top of a glass slide under anaerobic conditions. The XRD specimens were prepared similarly, but the final slides were finished with a layer of grease on top of the dried particle sample to protect the samples from oxidation. In the case of TEM sample preparation, 5 µL of anoxic water was added to the gold grid with ultrathin carbon film, and then, 10 µL of particle suspension was added. The XPS spectra were collected using a PHI Quantera SXM (ULVAC-PHI, Japan) with a hemispherical energy analyzer and a monoenergetic X-ray source (Al Kα: 1486.6 eV). The survey spectra were collected at 25 W/15 kV with a spot size of 100  µm, 45° take-off angle, and 280  eV pass energy. A 69 eV pass energy with 0.125 eV scan step was chosen for high-resolution spectrum acquisition. The high-resolution XPS spectra were fitted using Multipak software, with the charge correction based on adventitious C 1 s at 284.8 eV. The XRD samples were analyzed using a Rigaku MiniFlex II Desktop X-ray Diffractometer, which operates using Cu-tube Ka radiation at 30kV and 15mA at a scan rate of 1.5 degrees/minute. The TEM data were gathered using a JEOL JEM 2100 S/TEM at the Nanoscale Characterization and Fabrication Laboratory located at Virginia Polytechnic Institute and State University. The instrument was operated at 200 kV, and TEM bright field images were taken using a Gatan Ultrascan 1000XP CCD camera. The collection of selected area electron diffraction patterns was performed utilizing a Gatan Orius 833 slow-scan CCD camera. Furthermore, scanning TEM (STEM) mode was used to obtain Energy dispersive X-ray spectroscopy (EDS) data using a JEOL genuine 60 mm2 Silicon Drift Detector.

## RESULTS

### Growth profiles

In positive controls, it takes ~120 h for the cell culture to reach the end of the logarithmic phase, yielding a cell density of ~9.4 × 10^6^ cells/mL, and the stable phase spans from 140 h to 400 h with comparable optical density at 600 nm (OD_600_) and pigmentation intensity throughout the period (Fig. 2). The cell density was estimated through correlating the OD_600_ and cell counting results. The “py” cell culture has a longer lag phase than the positive control and rose to a cell density of ~2.5 × 10^6^ cells/mL, about one-quarter of that of positive controls, at ~240 h. We have identified further (slower) growth for “py” cell cultures after the OD reached a local maximum (at ~240 h), and such growth lasted till ~550 h. The cell growth in “py” was also quantified using the samples’ DNA yields, showing a maximum of ~4 ng/µL at ~550 h, consistent with the OD data. Depletion of sulfide was recorded at ~120 h for positive controls, and the production of sulfate through sulfur oxidation reached a maximum of 0.7 mM (Fig. 2). For “py,” sulfide concentrations remained below the detection limit while sulfate reached up to ~20 µM within the monitored period of up to 1000 h. The soluble iron concentrations in “py” showed a spike at ~550 h. The timing of the spike resonates strongly with that of maximum OD and DNA yield. It is noted that the maximum level of iron concentrations in “py” is still rather low, ~600 ppb, compared with that (~ 200–300 ppb) in the controls.

### Transcriptomic sequencing and differential gene expression analysis

**Fig 3:**
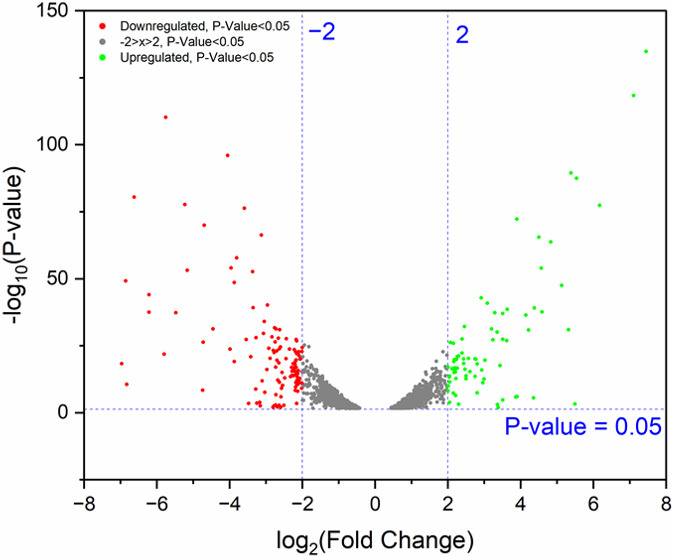
The volcano plot showing differential gene expressions in *A. vinosum* grown on pyrite versus dissolved sulfide. We used the log_2_FC < −2 or log_2_FC > 2 as the cutoff; the upregulated genes are displayed as green dots and downregulated genes as red dots.

The genome for *A. vinosum* has been reported to be 3.8 Mb encoding ~3,300 proteins and a similar number of genes (18). The transcriptomic sequencing analysis of the “py” and positive control samples identified a total of 3,302 genes, in line with the previous report. Through differential gene expression analysis of “py” vs. positive controls, and using log_2_FC > 2 or log_2_FC <-2 as well as *P* < 0.05 as the cutoff, we have identified a total of 80 upregulated and ~100 downregulated genes (Fig. 3; Table 1). Among these top differentially regulated genes, ~15% of the upregulated and 7% of the downregulated are associated with redox-active proteins such as cytochromes, hydrogenases, reductases, and others with Fe-S motifs. Sulfur metabolic genes accounted for 2% of the upregulated and 6% of the downregulated (using log_2_FC > 2 or log_2_FC <-2 as the cutoff). Genes associated with signal transduction and transcription regulation accounted for 8% of the upregulated and 3% of the downregulated. Photosynthetic RC-related genes were exclusively downregulated (except for those associated with carotenoid biosynthesis), accounting for 9% of the downregulated sequences. Interestingly, 3% of the upregulated genes are associated with metal efflux controls, and 4% (also of the upregulated) are concerned with cellular appendage sequences, including flagella, fimbriae, and pilin genes.

**TABLE 1:** Compilation of top differentially regulated genes for *A. vinosum* when grown on pyrite versus dissolved sulfide^*a*^

No.	Gene locus	log2FC	*P*_adj_	KEGG or Strindb annotation
Upregulated genes				
1	Alvin_1093	7.45	5.6E-132	Diheme cytochrome subunit of sulfide dehydrogenase
2	Alvin_0022	7.10	7.8E-116	Domain of unknown function DUF1924
3	Alvin_1092	6.18	1.7E-75	Flavocytochrome c sulphide dehydrogenase
4	Alvin_0023	5.54	1.8E-85	Diheme cytochrome c
5	Alvin_1379	5.50	1.9E-03	2-Isopropylmalate synthase
6	Alvin_1095	5.39	2.1E-87	Epoxyqueuosine reductase
7	Alvin_0024	5.32	8.9E-30	Membrane protein-like protein
8	Alvin_0021	5.13	4.6E-46	Cytochrome B561
9	Alvin_1094	4.83	4.2E-62	Uncharacterized protein
10	Alvin_0013	4.59	3.0E-36	Uter membrane efflux protein
11	Alvin_2309	4.57	2.1E-52	Hydrogenase (NiFe) small subunit HydA
12	Alvin_2308	4.50	7.3E-64	Hydrogenase (NiFe) small subunit HydA
13	Alvin_0020	4.38	1.0E-37	Diheme cytochrome c
15	Alvin_0014	4.21	1.1E-29	Efflux transporter, RND family, MFP subunit
16	Alvin_0025	4.15	3.8E-35	Two component transcriptional regulator
18	Alvin_2307	3.90	1.9E-70	Ni/Fe-hydrogenase, b-type cytochrome subunit
21	Alvin_2451	3.63	2.7E-37	Molybdopterin oxidoreductase Fe4S4 region
22	Alvin_1527	3.62	7.3E-26	FeoA family protein (Fe2 +transport)
23	Alvin_0019	3.51	2.4E-26	Ferrous-iron efflux pump FieF
26	Alvin_1848	3.51	1.1E-35	Isocitrate lyase
27	Alvin_2446	3.44	7.5E-17	Nitrite and sulphite reductase 4Fe-4S region
29	Alvin_1878	3.36	2.6E-03	Nitrogen fixation protein FixT
30	Alvin_0017	3.36	5.6E-29	XRE family transcriptional regulator
31	Alvin_2306	3.29	4.8E-36	Hydrogenase expression/formation protein, HoxM
32	Alvin_0018	3.24	4.1E-26	Di-heme cytochrome c peroxidase
33	Alvin_3291	3.20	4.8E-30	Hypothetical protein
34	Alvin_1145	3.09	1.9E-39	Periplasmic protein CpxP/Spy
35	Alvin_0015	3.02	9.0E-19	Heavy metal efflux pump, CzcA family
36	Alvin_2447	3.00	4.2E-12	Adenylylsulfate reductase
37	Alvin_2093	2.96	9.6E-11	Hydrogenase (NiFe) small subunit HydA
38	Alvin_0016	2.93	3.2E-15	Conserved hypothetical protein
39	Alvin_2111	2.92	1.9E-41	Sulfur-oxidizing protein SoxY
40	Alvin_3196	2.81	3.3E-07	Hypothetical protein
41	Alvin_0431	2.81	1.9E-17	Hypothetical protein
42	Alvin_1034	2.77	3.5E-19	Phosphoketolase
43	Alvin_3016	2.75	2.7E-19	Type IV fimbrial biogenesis protein FimT
44	Alvin_0929	2.75	4.8E-13	Hypothetical protein
45	Alvin_0926	2.61	9.7E-15	PRC-barrel domain protein
46	Alvin_2452	2.57	2.1E-19	Formate dehydrogenase, alpha subunit
47	Alvin_2092	2.50	3.3E-13	Conserved hypothetical protein
48	Alvin_2110	2.50	3.3E-12	Peptidase M48 Ste24p
49	Alvin_1143	2.47	1.4E-15	Twin-arginine translocation pathway signal
50	Alvin_1556	2.46	5.9E-31	Hypothetical protein
51	Alvin_3275	2.44	2.9E-19	Phage recombination protein Bet
52	Alvin_1525	2.43	1.1E-17	Ferrous iron transport protein B
53	Alvin_2112	2.40	2.4E-21	SoxZ; PFAM: Sulphur oxidation protein SoxZ
54	Alvin_1420	2.39	1.9E-26	Iron-sulfur cluster assembly transcription factor IscR
55	Alvin_1524	2.30	2.3E-03	Protein of unknown function DUF1920
56	Alvin_0483	2.29	2.8E-20	Catalase/peroxidase HPI
57	Alvin_1152	2.26	3.8E-20	Uncharacterized conserved protein UCP029693
58	Alvin_2311	2.26	5.9E-16	Transaldolase
59	Alvin_1146	2.24	4.4E-19	Hypothetical protein
60	Alvin_1446	2.22	2.7E-12	Antitoxin HigA-1
61	Alvin_2710	2.22	2.6E-06	Hypothetical protein
62	Alvin_1954	2.21	1.2E-14	Flagellar protein FliS
63	Alvin_1856	2.20	5.6E-16	Fe(ii) trafficking protein yggx;
64	Alvin_1521	2.19	2.3E-15	Cu(i)/ag(i) efflux system periplasmic protein cusf;
65	Alvin_0492	2.18	6.2E-19	Conserved hypothetical protein
66	Alvin_2145	2.18	6.9E-16	Sulfide:quinone oxidoreductase
67	Alvin_0026	2.18	1.5E-14	Integral membrane signal transduction histidine kinase
68	Alvin_0900	2.17	2.1E-14	Hypothetical protein
69	Alvin_2989	2.17	2.8E-18	NAD(*P*)H dehydrogenase (quinone)
70	Alvin_1144	2.15	3.9E-07	CsbD family protein
71	Alvin_1953	2.15	6.9E-25	Flagellar hook-associated two domain protein
72	Alvin_1150	2.12	9.5E-16	Conserved hypothetical protein
73	Alvin_1952	2.07	3.6E-25	Flagellar protein FlaG
74	Alvin_2454	2.06	5.3E-11	Formate dehydrogenase subunit gamma
75	Alvin_1877	2.05	5.3E-04	4Fe-4S ferredoxin iron-sulfur binding domain protein
76	Alvin_0107	2.05	3.9E-13	Conserved hypothetical protein
77	Alvin_2704	2.04	1.1E-12	Conserved hypothetical protein
78	Alvin_2312	2.01	1.2E-09	Integral membrane protein TerC (tellurite resistance)
79	Alvin_0098	2.01	2.8E-16	Transcriptional regulator, GntR family
80	Alvin_1154	2.00	7.4E-08	Conserved hypothetical protein
Downregulated genes				
81	Alvin_0704	−6.85	1.2E-47	Antenna complex alpha/beta subunit
82	Alvin_0703	−6.83	3.3E-10	Hypothetical protein
83	Alvin_0705	−6.62	1.5E-78	Hypothetical protein
84	Alvin_1741	−6.22	3.7E-36	Hypothetical protein
85	Alvin_0706	−6.21	1.4E-42	Antenna complex alpha/beta subunit
86	Alvin_0709	−5.80	6.1E-21	Light-harvesting complex one beta chain
87	Alvin_0962	−5.75	6.5E-108	Uncharacterized protein
88	Alvin_1740	−5.48	4.9E-36	Dinitrogenase iron-molybdenum cofactor biosynthesis protein
89	Alvin_2136	−5.23	9.4E-76	Hypothetical protein
90	Alvin_1739	−5.16	1.2E-51	Cobyrinic acid ac-diamide synthase
91	Alvin_1365	−4.74	4.0E-08	Ribulose-bisphosphate carboxylase
92	Alvin_1251	−4.72	2.5E-25	Dissimilatory sulfite reductase alpha subunit
93	Alvin_3072	−4.69	3.2E-68	Conserved hypothetical protein
94	Alvin_1252	−4.45	4.8E-30	Dissimilatory sulfite reductase beta subunit
95	Alvin_2515	−4.06	9.3E-94	Hypothetical protein
96	Alvin_2248	−3.99	8.1E-23	Outer membrane receptor for ferrienterochelin and colicins;
97	Alvin_2497	−3.96	1.7E-52	Conserved hypothetical protein
98	Alvin_0747	−3.87	2.6E-18	Peptidase C39 bacteriocin processing
99	Alvin_1006	−3.87	3.6E-47	Peroxiredoxin
100	Alvin_2498	−3.59	1.6E-74	Nitrogen fixation-related protein
101	Alvin_1253	−3.54	3.2E-26	DsrE
102	Alvin_1367	−3.48	1.3E-03	CbbQ/NirQ/NorQ domain protein
103	Alvin_0707	−3.41	5.2E-20	Regulatory protein LuxR
104	Alvin_2767	−3.37	4.1E-51	DEAD/DEAH box helicase domain protein
105	Alvin_1738	−3.35	7.8E-38	Cobyrinic acid ac-diamide synthase
106	Alvin_1711	−3.28	1.8E-08	Hypothetical protein
107	Alvin_0500	−3.27	6.8E-27	Protein of unknown function DUF150
108	Alvin_1366	−3.25	1.0E-03	Ribulose-bisphosphate carboxylase
109	Alvin_0749	−3.17	5.2E-04	Hypothetical protein
110	Alvin_2759	−3.15	8.0E-03	Hypothetical protein
111	Alvin_1841	−3.13	1.3E-64	Protein of unknown function
112	Alvin_2550	−3.10	2.2E-11	Antenna complex alpha/beta subunit
113	Alvin_2572	−3.07	2.0E-28	RNA polymerase, sigma 32 subunit, RpoH
114	Alvin_3032	−3.04	8.0E-33	Conserved hypothetical protein
115	Alvin_0708	−3.03	2.3E-07	Hypothetical protein
116	Alvin_1737	−2.98	1.9E-15	Dinitrogenase iron-molybdenum cofactor biosynthesis protein
117	Alvin_2429	−2.95	8.1E-39	NADH-quinone oxidoreductase, B subunit
118	Alvin_1508	−2.92	4.9E-23	Sulfur relay protein, TusE/DsrC/DsvC family
119	Alvin_1254	−2.88	1.9E-19	DsrF
120	Alvin_2576	−2.80	1.9E-02	Antenna complex alpha/beta subunit
121	Alvin_0501	−2.79	2.6E-22	NusA antitermination factor
122	Alvin_2250	−2.78	1.5E-21	Biopolymer transport protein ExbD/TolR
123	Alvin_2577	−2.78	7.9E-03	Antenna complex alpha/beta subunit
124	Alvin_2428	−2.76	1.8E-30	NADH (or F420H2) dehydrogenase, subunit C
125	Alvin_2768	−2.74	2.1E-25	RNP-1 like RNA-binding protein
126	Alvin_1122	−2.73	2.5E-16	Conserved hypothetical protein
127	Alvin_2554	−2.73	4.3E-03	Antenna complex alpha/beta subunit
128	Alvin_1688	−2.73	1.5E-06	Antibiotic biosynthesis monooxygenase
129	Alvin_2430	−2.73	4.4E-30	NADH-ubiquinone/plastoquinone oxidoreductase chain 3
130	Alvin_3073	−2.70	4.2E-22	C4-dicarboxylate transporter/malic acid transport protein
131	Alvin_0834	−2.69	9.0E-19	NAD(*P*)(+) transhydrogenase (AB-specific)
132	Alvin_2549	−2.68	1.8E-02	Antenna complex alpha/beta subunit
133	Alvin_2249	−2.66	1.1E-26	MotA/TolQ/ExbB proton channel
134	Alvin_2251	−2.65	8.3E-18	Biopolymer transport protein ExbD/TolR
135	Alvin_2551	−2.64	5.0E-03	Photosynthetic reaction centre cytochrome c subunit
136	Alvin_2600	−2.63	7.8E-23	SirA family protein
137	Alvin_0744	−2.63	6.5E-11	Aigma54 specific transcriptional regulator, Fis family
138	Alvin_2432	−2.62	8.7E-30	Triosephosphate isomerase
139	Alvin_0805	−2.60	1.4E-23	2-Oxo-acid dehydrogenase E1 subunit, homodimeric type
140	Alvin_2579	−2.59	1.9E-02	Antenna complex alpha/beta subunit
141	Alvin_1687	−2.58	6.6E-06	ATP dependent RNA helicase
142	Alvin_2760	−2.57	9.1E-09	Antenna complex alpha/beta subunit
143	Alvin_2254	−2.56	2.2E-14	Conserved hypothetical protein
144	Alvin_2548	−2.54	7.5E-10	Antenna complex alpha/beta subunit
145	Alvin_2552	−2.51	5.5E-03	Photosynthetic reaction center, M subunit
146	Alvin_1712	−2.48	2.1E-12	Conserved hypothetical protein
147	Alvin_0316	−2.45	2.2E-19	Transketolase
148	Alvin_2280	−2.44	1.7E-26	Translation initiation factor IF-1
149	Alvin_2599	−2.35	8.7E-23	Rhodanese domain protein
150	Alvin_1690	−2.32	6.1E-13	Transport system permease protein
151	Alvin_1754	−2.29	2.9E-13	Translation elongation factor *P*
152	Alvin_1689	−2.28	9.4E-15	Periplasmic binding protein
153	Alvin_2484	−2.25	1.5E-17	16S rRNA processing protein RimM
154	Alvin_1483	−2.24	2.9E-13	Hydrolase, TatD family
155	Alvin_1890	−2.23	2.1E-15	Acyl carrier protein
156	Alvin_0040	−2.20	3.1E-17	ATP synthase F0, A subunit
157	Alvin_2427	−2.20	2.7E-16	NADH dehydrogenase I, D subunit
158	Alvin_1691	−2.19	4.8E-08	ABC transporter related protein
159	Alvin_0499	−2.19	1.2E-14	Hypothetical protein
160	Alvin_1259	−2.19	1.7E-18	DsrL
161	Alvin_1753	−2.18	8.6E-22	tRNA synthetase class II
162	Alvin_1893	−2.18	3.3E-09	3-Oxoacyl-(acyl-carrier-protein) synthase III
163	Alvin_1258	−2.17	2.9E-26	dsrK
164	Alvin_1896	−2.17	7.0E-19	Protein of unknown function DUF177
165	Alvin_3195	−2.16	1.2E-03	Hypothetical protein
166	Alvin_0039	−2.16	3.3E-21	ATP synthase I chain
167	Alvin_0804	−2.15	9.7E-26	Pyruvate dehydrogenase complex dihydrolipoamide
168	Alvin_0746	−2.15	4.3E-10	Hypothetical protein
169	Alvin_0315	−2.14	6.4E-13	Glyceraldehyde-3-phosphate dehydrogenase, type I
170	Alvin_1734	−2.12	9.5E-08	Protein of unknown function DUF2269, transmembrane
171	Alvin_1260	−2.11	4.5E-15	dsrJ
172	Alvin_2426	−2.11	2.6E-10	NADH-quinone oxidoreductase, E subunit
173	Alvin_2758	−2.10	7.8E-22	Poly(A) polymerase
174	Alvin_2601	−2.09	2.0E-20	Conserved hypothetical protein
175	Alvin_2156	−2.08	6.6E-11	GTP-binding protein Obg/CgtA
176	Alvin_2252	−2.07	3.1E-12	TonB family protein
177	Alvin_2491	−2.06	3.8E-19	Molybdopterin oxidoreductase
178	Alvin_2602	−2.03	1.6E-19	Acetolactate synthase, large subunit
179	Alvin_2415	−2.01	8.4E-09	Conserved hypothetical protein
180	Alvin_1644	−2.01	1.4E-14	Integration host factor, beta subunit
181	Alvin_1079	−2.01	2.0E-10	Cytochrome B561
182	Alvin_2386	−2.00	2.2E-23	Peptide chain release factor 1

^*a*^ The genes without a designated annotation are highlighted in gray. (The complete data are in Table S2.)

Among the most differentially regulated genes, we identified a collection of cytochrome-related genes, whose fold change for the upregulated ones is up to ~200. For example, Alvin_1092 and Alvin_1093, which encode flavocytochrome *a* and *b*, involved in hydrogen sulfide-dependent cytochrome *c* reduction, are upregulated by up to 175-fold, and Alvin_0020 and Alvin_0023, which encode a diheme cytochrome *c*, are upregulated by ~47-fold. Others in the upregulated list include Alvin_0021, encoding a cytochrome *b*561 (which is in the region dominantly encoding *c*-type cytochromes), Alvin_2307, encoding a Ni/Fe hydrogenase *b*-type cytochrome subunit, Alvin_2452–2454, encoding three formate dehydrogenase subunits, and Alvin_2989, encoding NAD(*P*)H dehydrogenase. The downregulated genes, excluding those tabulated in Tables 1 to 3 (which will be discussed in the following paragraphs), include several genes related to dehydrogenases found in carbon metabolism cycles, such as Alvin_0315, Alvin_0804–805, and Alvin_2427–2428. The former three encode glyceraldehyde-3-phosphate dehydrogenase, pyruvate dehydrogenase complex dihydrolipoamide, and 2-oxoacid dehydrogenase E1 subunit, respectively, whereas the latter two encode NADH dehydrogenase subunits.

**TABLE 2:** Differential expression of *puf* and *puc* genes in the pyrite-supported *A. vinosum* cells^*a*^

Gene	Protein	log_2_FC	*P*_adj_
*puf* genes (LH1)			
Alvin_2550	puf/LH1	−3.105	2.23E-11
Alvin_2554	puf/LH1	−2.729	0.0042904
Alvin_2549	puf/LH1	−2.685	0.0180326
Alvin_2551	pufC	−2.637	0.0050426
Alvin_2548	puf/LH1	−2.543	7.53E-10
Alvin_2552	pufM	−2.506	0.0054761
Alvin_2555	puf/LH1	−1.963	9.55E-05
Alvin_2553	pufL	−1.657	1.18E-06
Alvin_2634	puf/LH1	−1.571	1.35E-11
*puc* genes (LH2)			
Alvin_0704	pucB6	−6.853	1.15E-47
Alvin_0703	pucA6	−6.829	3.27E-10
Alvin_0705	pucA5	−6.622	1.51E-78
Alvin_0706	pucB5	−6.214	1.40E-42
Alvin_0709	pucB4	−5.797	6.10E-21
Alvin_2759	pucA3	−3.155	7.98E-03
Alvin_0708	pucA4	−3.027	2.25E-07
Alvin_2576	pucA2	−2.803	1.89E-02
Alvin_2577	pucB2	−2.775	0.0079294
Alvin_2579	pucB1	−2.594	1.93E-02
Alvin_2760	pucB3	−2.570	9.14E-09
*Alvin_2578*	pucA1	−1.997	0.000097

^*a*^ Only those with a *P* value of <0.05 are presented.

**TABLE 3:** Differential expression of *sox* and *dsr* genes in the pyrited-supported *A. vinosum* cells^*a*^

Gene	Protein	log_2_FC	*P*_*adj*_
*dsr* genes			
Alvin_1251	DsrA	−4.723	2.54E-25
Alvin_1252	DsrB	−4.454	4.76E-30
Alvin_1253	DsrE	−3.541	3.15E-26
Alvin_1254	DsrF	−2.883	1.89E-19
Alvin_1259	DsrL	−2.187	1.70E-18
Alvin_1258	DsrK	−2.168	2.94E-26
Alvin_1260	DsrJ	−2.113	4.47E-15
Alvin_1261	DsrO	−1.987	5.20E-20
Alvin_1262	DsrP	−1.678	1.86E-15
Alvin_1256	DsrC	−1.247	1.07E-07
*sox* genes			
Alvin_2111	SoxY	2.919	1.91E-41
Alvin_2112	SoxZ	2.40	2.39E-21
Alvin_2167	SoxB	1.22	2.19E-07
Alvin_2169	SoxA	1.175	0.00714587
Alvin_2168	SoxX	1.074	0.00178113

^*a*^ Only those with a *P* value of <0.05 are presented.

The genes encoding metal ion transporters, Na^+^/H^+^ antiporter, and flagella, fimbriae, and pili components are also in the most differentially regulated list. For metal ion transporters, Alvin_0013–0015 likely represent components of efflux transporters of the RND and CzcA families, and Alvin_0019 and Alvin_1521 are, respectively, associated with FieF Iron efflux pump and a periplasmic efflux protein. The upregulated flagella-associated genes include Alvin_1952–1954, encoding FlaG, a flagellar hook-associated protein, and FliS, respectively. Alvin_3016 is associated with fimbriae biogenesis (i.e., FimT), and Alvin_1186 with a pilin protein PilT.

The expression of genes associated with light harvesting and dissimilatory sulfur metabolism pathways showed consistently distinct patterns for “py” versus positive controls (Tables 2 and 3; Fig. S1 and S2). In the case of light-harvesting complexes, genes relevant to the biosynthesis of LH1, LH2, and reaction center components were exclusively downregulated. Specifically, the gene clusters, *pufC*, *pufM*, and *pufL*, which are co-transcribed with three sets of *pufA* and *pufB* genes, encoding LH1 apoproteins, were suppressed by various levels, up to 10-fold (Alvin_2547–2555). The upstream *pufH* along with adjacent genes, encoding photosynthetic complex assembly proteins and a hypothetical protein, was also slightly suppressed (Alvin_2634–2637). The genes associated with LH2 apoproteins were suppressed the most, by up to 115-fold (Alvin_0703–0706, and 0708–0709). By comparison, genes related to the biosynthesis of Bchl*a* and carotenoids were either moderately suppressed or enhanced in expression (Alvin_1182–1183, 2556, 2561–2563, 2638–2643, and 2564–2570). In the case of dissimilatory sulfur metabolism, we evaluated the expression of three genes related to Sgp proteins and found upregulation of Alvin-1095 (representing SgpA) by ~42-fold in the differential analysis of “py” vs. positive controls. The other two genes, Alvin_0358 and Alvin_1325, were either slightly downregulated or unchanged. By comparison, *dsr* genes are exclusively downregulated except for *dsrC*. Specifically, *dsr*A/Alvin_1251 and *dsr*B/Alvin_1252, which form a *dsrAB* complex, show a ~ 22-fold expression suppression. The other complex within the *dsr* loci is *dsrEFH*, from which *dsrE*/Alvin_1253 decreases by 11-fold and *dsrF*/Alvin_1254 by 7-fold. The genes coding membrane-bound Dsr proteins were also downregulated, by ~4-fold for *dsrJ/*Alvin_1260, 4-fold for *dsrO*/Alvin_1261, and 3-fold for *dsrP/*Alvin_1262. The only gene that remained relatively unchanged in its expression level is *dsrC*/Alvin_1256. Besides *dsrC*, there are four more genes annotated as TusE/DsrC/DsvC family sulfur relay proteins, namely Alvin_0028, Alvin_0345, Alvin_0732, and Alvin_1508^18^, which are, respectively, upregulated by ~2-, ~2-, and ~4-fold and downregulated by ~7-fold. In the *sox* loci, genes encoding SoxYZ complex were upregulated by 5-fold, whereas the rest of the *sox* genes seemed unaffected in terms of expression levels.

It is important to highlight that a considerable portion of the highly upregulated/downregulated genes, ~30% of the upregulated and ~23% of the downregulated genes, were considered hypothetical proteins or domains of unknown function (DUF) (Table S1). A taxonomy analysis was carried out to infer how prevalent and conserved the sequences of these genes might be within γ-Proteobacteria. In the taxonomy analysis, the percentage of hits from reported γ-Proteobacteria sequences among the total hits was presented, indicative of the potential relationship of the unknown protein to γ-Proteobacteria. The analysis shows that 14 out of 18 upregulated genes and 16 out of 25 downregulated hypothetical sequences show ~90% or higher Basic Local Alignment Search Tool (BLAST) results. We have also identified certain motifs (e.g., signal peptides) and transmembrane domains in some of these unknown protein sequences.

### Pyrite substrate analysis

**Fig 4:**
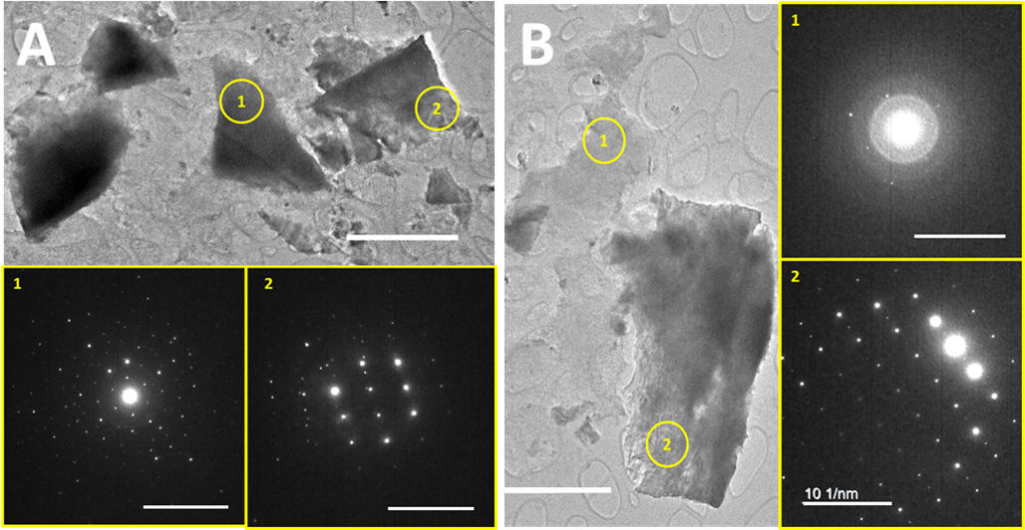
HR-TEM displaying plate-like fragments of the solid substrate recovered from *A. vinosum*-pyrite culture medium at the end of the experiments t > 1,000 h. The solid materials from the cell culture consist of a significant fraction of amorphous phases (**B1**), distinctive from the abiotic controls. The biological samples may contain pyrrhotite (FeS), elemental sulfur (S), and an unknown amorphous phase beside pyrite based on d-spacing estimation using the obtained electron diffraction micrographs.

**Fig 5:**
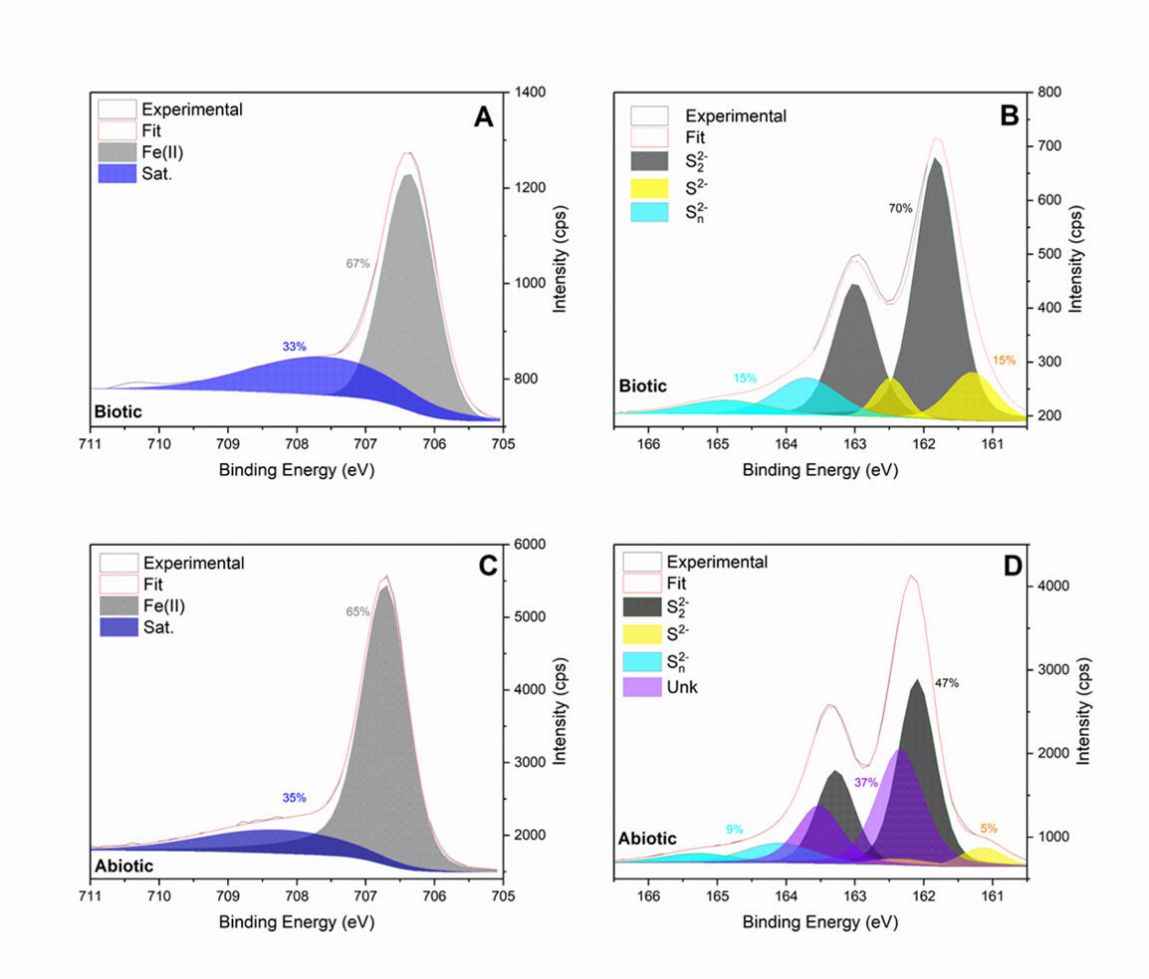
X-ray photoelectron spectroscopy (XPS) analysis of iron and sulfur speciation for samples and controls (recovered at the end of the experiments t > 1,000 h). (**A**) Iron spectra for biological pyrite-*A. vinosum* samples. (**B**) Sulfur spectra for biological pyrite-*A. vinosum* samples. (**C**) Iron spectra for abiotic pyrite controls. (**D**) Sulfur spectra for abiotic pyrite controls.

The pyrite recovered from the cell culture medium showed irregularly shaped particles with wide-ranging dimensions of ~100 nm to several microns (µm) (Fig. 4). In these biological pyrite samples, we observed apparent amorphous domains, with no electron diffraction patterns and richer in sulfur compared with the highly crystalline domains. Based on the d-spacings obtained using the electron diffraction micrographs, the solids in the *A. vinosum* culture consist of pyrite, and likely pyrrhotite and elemental sulfur. Although different interpretations may be made based on the electron diffraction patterns alone, the corresponding XPS analyses provide extra constraints on the Fe and sulfur valence states and bonding as well as sulfur-to-iron composition ratios (Fig. 5). The results showed that only Fe(II) was present in both abiotic and biotic pyrite samples. An asymmetric fit was performed on the iron region of the spectra to calculate the relative abundance of the species identified. The abiotic control (pyrite) showed a main 2p3/2 peak at 706.72 eV, matching the binding energy for Fe(II) valence electrons in pyrite, along with a satellite peak at 707.95 eV, likely resulting from surface defects. The “py” sample (biotic sample) had a peak at 706.37 eV in the iron region, with a satellite peak at 707.58 eV, which may indicate the presence of Fe(II)-O speciation (i.e., adsorption of soluble Fe(II) on solid surfaces). Both samples displayed the same oxidation state of Fe(II), with no evidence of Fe(III) and its satellite peak. The surface composition of both materials was extremely similar, with a small increase in the dominance of the Fe(II) 2p3/2 peak, from 65% in the control to 67% in the biotic sample. The fit of the sulfur region spectra required doublet peaks, with the area of the 2p3/2 peak set to be twice that of the p1/2 and the distance between them set at 1.18 eV. The abiotic control showed four different sulfur species, including S^2-^ at 161.14 eV for p3/2, with an orbital split p1/2 peak at 162.32 eV, polysulfide at 164.1 eV for p3/2, with a p1/2 peak at 165.28 eV, disulfide at 162.1 eV for p3/2, with a p1/2 peak at 163.28 eV, and a fourth unidentified peak at 162.34 eV and matching orbital split at 163.52 eV. The fourth peak fell 0.14 eV away from the main disulfide peak, but its corresponding species is unknown. The biotic sample showed three sulfur species, including S^2-^ at 161.3 eV and 162.48 eV, disulfide at 161.82 eV and 163 eV, and polysulfide at 163.7 eV and 164.88 eV. Both the control and biotic pyrite samples showed the presence of monosulfide, disulfide, and polysulfide. However, the unidentified peak close to the main disulfide peak in the control disappeared in the biotic sample. Upon closer comparison, the biotic sample showed increases in the abundance of polysulfides from 9% in the control to 15% in “py,” of monosulfide from 5% to 15% and of disulfide from 47% to 70%. Nevertheless, considering that apical, bridging, and terminal ligands cause a significant peak position shift, and including the unknown 0.14 eV peak as a variation of disulfide, the biotic sample disulfide decreased from 85% in the control to 70% overall. The relative abundances of each iron/sulfur species were estimated based on the XPS analysis, and interestingly, the overall sulfur-to-iron ratio increased greatly for the “py” samples, reaching ~12.6, compared with that for the negative control samples, ~3.9.

## DISCUSSION

The cell growth profiles and transcriptomic analysis results revealed significant changes in the cells’ major metabolic pathways, including electron transport, RC and LH complex biosynthesis, and sulfur oxidation. We have specifically discussed these changes in the following section. Combining these molecular biological analyses with the pyrite substrate analyses, we have also proposed mechanisms of interaction between *A. vinosum* and pyrite that enabled the bacterial cells’ autotrophic growth.

### Key roles of cytochromes in *A. vinosum*-pyrite electron transfer

The genome of *A. vinosum* encodes a wide range of cytochromes that are known to play key roles as diffusible electron carriers, dissimilatory sulfur metabolism enzymes, hydrogenases, etc. In the current study, up to ~200-fold upregulation was identified for a number of genes related to *c*-type and, to a lesser extent, *b*-type cytochromes in the “py” cell cultures. Further analyses revealed that some of the upregulated genes are associated with soluble or membrane-bound *c*-type cytochromes or flavocytochromes (Alvin_1093, 0020, and 0023), previously classified as diffusible electron carriers. It is noted that Alvin_1093 is one of the top upregulated genes (expression increased by ~175-fold) in the “py” cells. Alvin_1093 and Alvin_1092 (upregulated by ~75-fold) encode a heterodimer consisting of a 21 kDa diheme cytochrome *c* subunit (FccA) and a 46 kDa flavin-binding subunit (FccB) in *A. vinosum* (4). Although soluble *c*-type cytochromes were shown to catalyze the oxidation of sulfide to sulfur or polysulfides *in vitro* (22), the roles of FccA and FccB in *A. vinosum* remain unresolved. As pointed out in Weissgerber *et al*. (18) mutants in which the genes *fccAB* are inactivated by a kanamycin cassette still oxidize sulfide with rates similar to the wild type (22). Some sulfide-utilizing green sulfur bacteria, for example, *Chlorobium luteolum*, and purple sulfur bacteria, for example, *Thiocapsa roseopersicina*, *Thiococcus pfennigii*, and *Allochromatium warmingii*, do not produce flavocytochrome *c*, which is an additional hint that flavocytochrome *c* is not essential for sulfide oxidation (4). Interestingly, Alvin_1093 along with Alvin_1092, 0020, and 0022–0023 showed distinctive regulation patterns for the pyrite-supported cells in this study than the elemental sulfur (S^0^)-supported cells (also of *A. vinosum* DSM180) in a previous study (59) (Table S2 and data sets). Specifically, Alvin_0020, 0022, and 0023 were significantly suppressed in the S^0^-supported, photoautotrophically grown cells versus their positive controls using soluble sulfide, whereas Alvin_1093 was slightly enhanced (59). Such evident variations strongly indicate that Alvin_0020, 0022–0023, and 1092–1093 have played particularly important roles in the *A. vinosum*-pyrite interactions in the current study (further discussion of Fcc is provided in the “dissimilatory sulfur metabolism” section). Up to 41-fold upregulation of Alvin_1095, associated with a 4-heme *c*-type cytochrome, was also observed although the component’s function and pathway have not been resolved.

We further evaluated whether cytochromes, especially those with multihemes, may play a role in the *A. vinosum* cell-pyrite electron transfer, linking intracellular energy reactions to the oxidation of solid pyrite external to the cells. The phenomenon of extracellular electron transfer (EET) has been demonstrated in over ~100 microbes to date, perhaps most notably in *Geobacter sulfurreducens* and *Shewanella oneidensis*, where a network of multiheme *c*-type cytochromes on the inner membrane, periplasm, and outer membrane couple intracellular energy reactions with the use of external solid electron donors or acceptors (60–63). Multiheme cytochromes (MHCs) in particular are key players in extracellular electron transfer (62), as the proximity and arrangement of hemes can allow efficient intraprotein electron transfer (64). We identified 43 putative *c*-type cytochromes in *A. vinosum* based on the presence of CXXCH heme *c* binding motifs, and of these, 18 were putative MHCs (containing multiple CXXCH motifs): specifically, 11 × diheme, 1 × 3 heme, 3 × 4 heme, 1 × 7 heme, and 2 × 8 heme cytochromes (Table S3). Some of the larger ones (e.g., 7- or 8-heme) in particular, and various others, have no annotated functions; the expression of these larger MHCs was exclusively enhanced in the “py” cells. The remaining 25 are putative monoheme *c*-type cytochromes. We also probed these genes for the presence of LXXC lipid-binding motifs and/or signal peptides, as both periplasmic and membrane-associated cytochromes are required for extracellular electron transfer. LXXC is a lipoprotein consensus sequence for signal peptidase II found in key outer membrane cytochromes in *S. oneidensis (*65). SignalP (66) can detect 5 types of signal peptides, that is, a protein can enter the cell’s secretory pathway, where it may be localized to the inner membrane, exported to periplasm, or localized to the outer membrane. In total, 19 out of 43 putative cytochromes contained LXXC lipid motifs, 21 were detected by SignalP, and eight were detected for both. The fact that multiple cytochromes are potentially associated with the inner or outer membrane (with others not identified here possibly being soluble electron carriers) is promising for identifying a potential cytochrome network for extracellular electron transfer in *A. vinosum*. Experimental evidence will be required to confirm the cellular localization of cytochromes in *A. vinosum*, and whether they contribute to extracellular electron transfer. As a disclaimer, other cytochromes of interest may exist, for example, those without heme *c* motif (CXXCH) or those not detected by the LXXC lipid motif or by SignalP. In total, 10 putative *c*-type cytochromes (including an 8-heme, 2 diheme, and 7 monoheme) were upregulated in the “py” cells and may be of particular interest toward investigating the coupling of carbon fixation at the inner membrane to the oxidation of pyrite outside the cell.

### Less important roles of LH and RC complex components?

**Fig 6:**
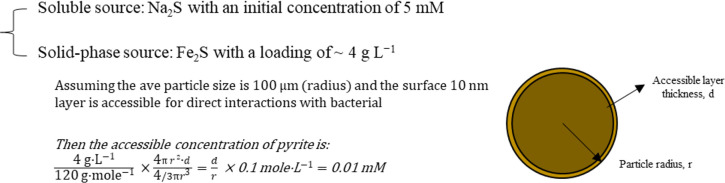
Comparison of the availability of solid-phase pyrite versus soluble Na_2_S as an electron donor.

Another major change identified in the “py” cells is the downregulation of photosynthetic genes related to the biosynthesis and assembly of LH and RC components (Table 2; Fig. S1). As a recap, the expression of *puc* clusters encoding LH2 apoproteins was significantly suppressed, by up to ~70-fold; the *puf* clusters and genes related to biosynthesis of Bchl *a* were also downregulated, by ~8- to 10-fold for the former and by ~2-fold for the latter. The only genes not affected or enhanced in expression within the photosynthetic category are those representing carotenoids biosynthesis (Alvin_2564–2570). It is still premature to conclude what has caused the extensive downregulation of the photosynthetic LH- and RC-related genes in the “py” cells. A most likely reason might be that the growth rate of the “py” cells was limited by the electron supply and its connection to the carbon fixation pathway, and thus, the demand for higher-density LH and RC complexes was no longer existent. We have compared the availability of solid-phase pyrite versus soluble Na_2_S as an electron donor (Fig. 6) assuming that the electron scavenge was restricted in the surface layer of pyrite and found ~2–3 order of magnitude difference in their effective concentrations.

It is noted that the relationships among the RC complex, sulfur-oxidation pathway, and carbon fixation pathway are not fully understood. In other words, it is unknown whether the reactivity of RC complex is specifically affected by the electron donor source. If so, the possibility of bypassing the LH-RC complex by the bacterial cells when using an alternative electron source cannot be ruled out. We further evaluated the expression of genes related to ribulose 1,5-biphosphate carboxylase/oxygenase (RuBisCO) in the “py” and positive control cells as both types grew autotrophically with bicarbonate as the sole carbon source. *A. vinosum* possesses two complete sets of genes encoding for RuBisCO subunits: the large subunit RbcA/RbcB represented by Alvin_1365–1366 and the small one RbcS/RbcL represented by Alvin_2749–2750 (67). Opposite trends have been observed for the two sets of RuBisCO genes in the “py” cells, with Alvin_1365–1366 downregulated by at least 10-fold and Alvin_2749–2750 moderately upregulated by ~2-fold. According to the gene arrangement, the *rbcAB* gene belongs to IAq-form RuBisCO genes, typically associated with *cbbQ* encoding proteins affecting RuBisCO activity (68), whereas the *rbcSL* genes are IAc-form RuBisCO genes (69). Besides the RuBisCO genes, *A. vinosum* harbors a gene encoding an IV-type RuBisCO-like protein (RLP) (Alvin_2545), the expression of which decreased just slightly in the “py” cells. It remains unclear what roles such RLPs play in *A. vinosum* metabolism, but likely not involved in RuBis-dependent CO_2_ fixation (70, 71).

**Fig 7:**
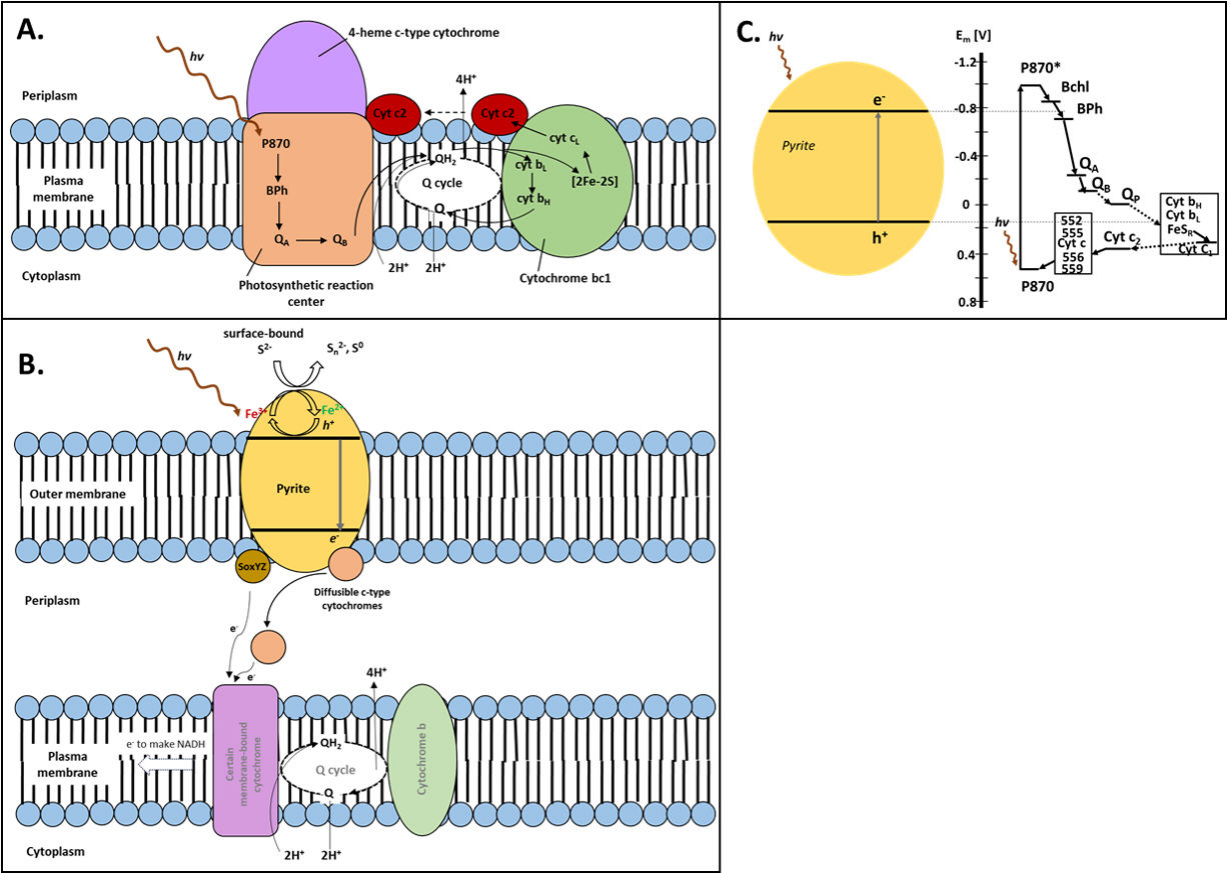
Proposed oxidation of pyrite driven by both photochemistry and diffusible and membrane-bound cytochromes in *A. vinosum*. (**A**) Illustration of major proteins and other components in PSB’s RC complex. (**B**) Proposed mechanisms for pyrite oxidation by *A. vinosum*. (**C**) Comparison of energy levels for PSB photosynthetic electron carriers vs. pyrite conduction/valence bands (potential values obtained from references 72, 73).

An alternative explanation for the probable “shutdown” of LH and RC, other than the electron donor restriction, might be that the cells have established a less “expensive” pathway for obtaining energy to drive their carbon fixation and growth. Regarding what other pathways may be possible for *A. vinosum* cells to capture light energy, here, we present a new hypothesis that requires further experimental evidence. In this hypothesis, we assume that the electron transfer from the extracellular pyrite substrate can be driven by both photochemical and non-photochemical reactions to support CO_2_ fixation, and these mechanisms do not involve RC complexes in *A. vinosum* (Fig. 7). There are obvious energy and nutrient appeals for *A. vinosum* to enable such cell-pyrite interactions, which are further discussed in the “hypothetical model” for pyrite oxidation by *A. vinosum*.

### Dissimilatory sulfur-oxidation metabolism

For genes encoding major enzymes (likely) involved in dissimilatory sulfur metabolism, we have observed opposite trends in their differential expressions (in “py” vs. positive control), primarily divided by associated pathways of the relevant enzymes (Table 3; Fig. S2). We will first discuss the genes representing Fcc and Sqr, respectively, although their roles in dissimilatory sulfur oxidation have not been fully resolved. It has been pointed out in our former discussion on cytochromes that FccA and FccB, the two subunits constituting an enzyme catalyzing sulfide oxidation and a cytochrome reduction in the periplasm, are likely key in enabling the *A. vinosum*-pyrite electron transfer. Chen *et al*. (74) provided a detailed illustration of Fcc structures, which consist of a glutathione reductase-like flavin-binding subunit and a diheme cytochrome subunit. Specifically, the diheme cytochrome folds as two domains with an unusual interpropionic acid linkage joining the two heme groups in the interior of the subunit, and a tryptophan, threonine, or tyrosine side chain may provide a partial conduit for electron transfer to one of the heme groups located ~10 Å from the flavin. This structural configuration of FccA or B cannot rule out the possibility of it bridging membrane-bound pyrite oxidation to periplasmic metabolisms other than oxidizing pyrite within the periplasmic space. Meanwhile, *A. vinosum* contains two membrane-bound Sqr enzymes belonging to types IV (Alvin_2145) and VI (Alvin_1195) (59). Sqr belongs to a family of FAD-dependent oxidoreductases utilizing a motif of Cys-S-S-Cys as the key redox site (75). Sqr has been previously identified to reduce the quinone pool present in the photosynthetic or plasma membranes of purple bacterial cells and was proposed as a candidate protein for oxidizing sulfide (22, 76). In the case of *Rhodobacter capsulatus*, polysulfides were identified as the main reaction products *in vitro*. In our current study, opposite trends were observed in the differential gene expressions (“py” versus positive control) for Alvin_2145, encoding type IV SqrD (upregulated by ~4.5-fold) and for Alvin_1195, encoding type IV SqrF (downregulated slightly). A correlation between the occurrence of SqrD and the production of intracellular sulfur globules has been suggested previously (23) mainly through observations that *sqrD* genes are present in members of Chromatiaceae but absent in species of Ectothiorhodospiraceae that exclusively produce extracellular sulfur globules. Relevant to this discussion, we identified sulfur-rich amorphous phase in the biologically reacted pyrite in the TEM analysis. However, we have not confirmed the source of this possibly polymeric sulfur phase, that is, whether it was intracellular or pyrite oxidation product. The downregulation of Alvin_1195 is consistent with the previous understanding that SqrF is involved in optimizing cell growth at high sulfide concentrations (23), which was not the case for the “py” cell cultures in this study. It is still unknown if the cells grown on pyrite in this study can form sulfur globules in the periplasm. The genes representing the envelope proteins of such sulfur globules (i.e., SgpA, SgpB, and SgpC) showed interesting patterns in differential gene expression analysis, however. Specifically, SgpA, SgpB, and SgpC are encoded by Alvin_1905, Alvin_0358, and Alvin_1325, respectively. SgpC plays an important role in globule expansion, whereas SgpA and SgpB can be replaced by each other to some extent (77, 78). In our study, Alvin_1905 and Alvin_0358 were slightly downregulated, and Alvin_1325 remained unchanged. We note here that the expression of genes representing Sgp was not apparently suppressed in the “py” cell cultures compared with positive controls, which creates a sharp contrast with the trends previously reported for “S^0^-supported” cells (59).

The general trend for the three clusters of *sox* genes is moderately upregulated or relatively unaffected in the “py” cells (compared with positive controls). It is noted that the Sox protein complex is localized in the periplasm, which differs from the location of Dsr proteins. Although Dsr proteins were implicated as key participants in the oxidation of sulfur globules, genes related to Dsr are downregulated in the current study [except that *dsrC* (Alvin_1256) remained relatively unchanged in its expression level]. In fact, a review chapter on dissimilatory sulfur metabolism in purple sulfur bacteria pointed out that purple non-sulfur bacteria, including those able to oxidize elemental sulfur lack *dsr* genes (28) and the *A. vinosum* cells grown upon external sulfur, showed significant downregulation in their *dsr* genes (59). Combined with the latest results in this study, it is strongly suggested that Dsr are not highly involved in the metabolism of external solid substrates of sulfur. Dsr proteins are largely localized in the cytoplasm, with a transmembrane complex (DsrMKJOP). It is likely that the specific locality and connection to photosynthetic electron transport chains (79) of Dsr proteins make it difficult for most of them to participate in pyrite utilization if pyrite oxidation occurred largely outside the cells, and the produced intermediate sulfur species differed from those produced through soluble sulfide oxidation. It is noted that although the *dsr* genes are transcribed as one single element, *dsrC* has an additional independent promoter site (80), pointing to a special function of DsrC. Furthermore, besides *dsrC*, there are four more genes annotated as TusE/DsrC/DsvC family sulfur relay proteins, namely Alvin_0028, Alvin_0345, Alvin_0732, and Alvin_1508. We observed upregulation by ~2-fold to 4-fold for Alvin_0028, 0345, and 0732 and downregulation by ~8-fold for Alvin_1508.

The *dsr* gene expression data are consistent with the lack of sulfate in the “py” cell culture medium (i.e., IC data), both of which suggest that *A. vinosum* cells might be capable of oxidizing pyrite (or specifically pyrite surface-bound sulfur) to polysulfide or elemental sulfur, but not able to further oxidize these sulfur species to sulfate. However, we also note that pyrite is the sole sulfur source for the “py” cells, which may assimilate any sulfate produced from the bacterial oxidation of pyrite. Further comparative kinetic studies are necessary to verify if *A. vinosum* can oxidize pyrite to sulfate.

### Information from flagellum, fimbriae, and pilin genes

We have singled out the genes associated with the biosynthesis of flagella, fimbria, and pili because the expression of these genes was exclusively enhanced in “py.” Many species of purple bacteria swim with the assistance of flagella toward carbon/other nutrient sources and light, using a complex set of chemosensory pathways (81). The flagellum in bacterial cells is an extremely complex structure, requiring the expression of genes encoding flagellar proteins to be tightly regulated and ordered. The upregulation of Alvin_0408, 1188, 1569, and 3021 opens a discussion on whether flagella, fimbria, and pili are involved in establishing physical contact between *A. vinosum* cells and pyrite. Furthermore, although a possible connection between flagellation and substrate exploration and utilization has not been shown in bacterial cells, flagellar proteins were recently speculated to be involved in direct physical contact with insoluble elemental sulfur for oxidation in *Aquifex aeolicus* (82). Overall, the extensive upregulation of flagellum-, fimbriae-, and pilin-related genes manifests two key messages. First, mobility may be a critical factor for *A. vinosum* cells grown upon pyrite. High mobility may help the cells to move around easily to either find the most “bioavailable” spots on pyrite or avoid the potential cytotoxic effects of substrate surface radical species (which are common in photochemical reactions) and oxidation products. Second, the enhanced expression of appendage genes also indicates that physical contact is likely important in cell-pyrite interactions.

### Hypothetical model for pyrite oxidation by *A. vinosum*

Based on the obtained solution, substrate, and gene expression analyses, we have proposed a hypothetical model for pyrite oxidation by *A. vinosum* (Fig. 7). In this model, physical contact of bacterial cells and pyrite particle surfaces is necessary for the pyrite-supported cell growth. The utilization of pyrite is proposed to be driven by both photochemical and non-photochemical processes. As pyrite has a band gap of 0.9 eV (83), the illumination setup for the experiments is capable of exciting the charge separation in pyrite. Certain monoheme *c*-type cytochromes may play a role as diffusible electron carriers, leading to the oxidation of surface-bound sulfur. Meanwhile, the periplasmic proteins SoxY and SoxZ may bind to the sulfur on pyrite surfaces and catalyze their oxidation. Both SoxYZ and diffusible electron carriers will pass the electrons to a membrane-bound *c*-type cytochromes, which relay the electrons through a quinone pool to cytochrome *b* to subsequently generate adenosine triphosphate (ATP). The various cytochromes involved in the proposed pathways are yet to be identified, but from the upregulated list (based on the gene expression analyses), several candidates with compatible reduction potentials may fit into these roles. It is noted that there is no evidence that *A. vinosum* is capable of oxidizing ferrous iron (separately tested in the current study). This hypothetical model well explains the cryptic behavior of dissolved iron in the solution as the initial charge separation in pyrite is more likely to oxidize structural Fe(II) to Fe(III), subsequently oxidizing the sulfur while being reduced back to Fe(II); these cyclic reactions may lead to iron mobilization and/or monosulfide reprecipitation depending on the locality of the sulfur involved in the process. Further experimental evidence is required to validate this hypothetical model.

### Conclusion

In this study, we showed that *A. vinosum* cells are capable of autotrophic growth using pyrite as the source of sulfur and electron donors. The differential gene expression analysis along with growth profile and substrate characterization data provided valuable insight into the molecular mechanisms underlying the bacterial autotrophic growth. Up to ~200-fold upregulation of genes encoding for a range of c-type and b-type cytochromes (including multiheme ones) points to the high relevance of these proteins in scavenging and relaying electrons from pyrite to key metabolic pathways. Conversely, the exclusive downregulation of LH and RC complex components may suggest that the available electron donor source likely has dominant control over the bacterial cells’ photosynthetic activity. The possibility that *A. vinosum* may bypass some or all of the photosynthetic pathway and couple the electron scavenging from pyrite directly to carbon fixation is not ruled out. The results of this study have, for the first time, put the interplay of purple sulfur bacteria and transition metal sulfide chemistry under the spotlight, with the potential to advance multiple fields, including metal and sulfur biogeochemistry, bacterial extracellular electron transfer, and artificial photosynthesis.

## SUPPLEMENTAL MATERIAL

Fig. S1 aem.00863-24-s0001.tif Schematic illustrating locations of LH1, LH2, and RC complexes as well as their recognized role in sulfur oxidation.

Fig. S2 aem.00863-24-s0002.tif Schematic illustrating locations of Sox and Dsr proteins and their recognized role in sulfur oxidation.

Supplemental material aem.00863-24-s0003.pdf Ingredients of mineral mix, and graph of pH for control and pyrite cell culture media.

Table S1 aem.00863-24-s0004.xlsx Upregulated and downregulated genes.

Table S2 aem.00863-24-s0005.xlsx Pyrite versus elemental sulfur.

Table S3 aem.00863-24-s0006.pdf Compiled list of 43 putative c-type cytochromes in *A. vinosum* and their corresponding gene expression status in the pyrite culture samples.

ASM does not own the copyrights to Supplemental Material that may be linked to, or accessed through, an article. The authors have granted ASM a non-exclusive, world-wide license to publish the Supplemental Material files. Please contact the corresponding author directly for reuse.
