# Supplementary figures and images for: Evidence for autotrophic growth of purple sulfur bacteria using pyrite as electron and sulfur source
Source: Appl Environ Microbiol. 2024 Jun 20;90(7):e00863-24. doi: 10.1128/aem.00863-24 (PMC11267869; doi:10.1128/aem.00863-24)

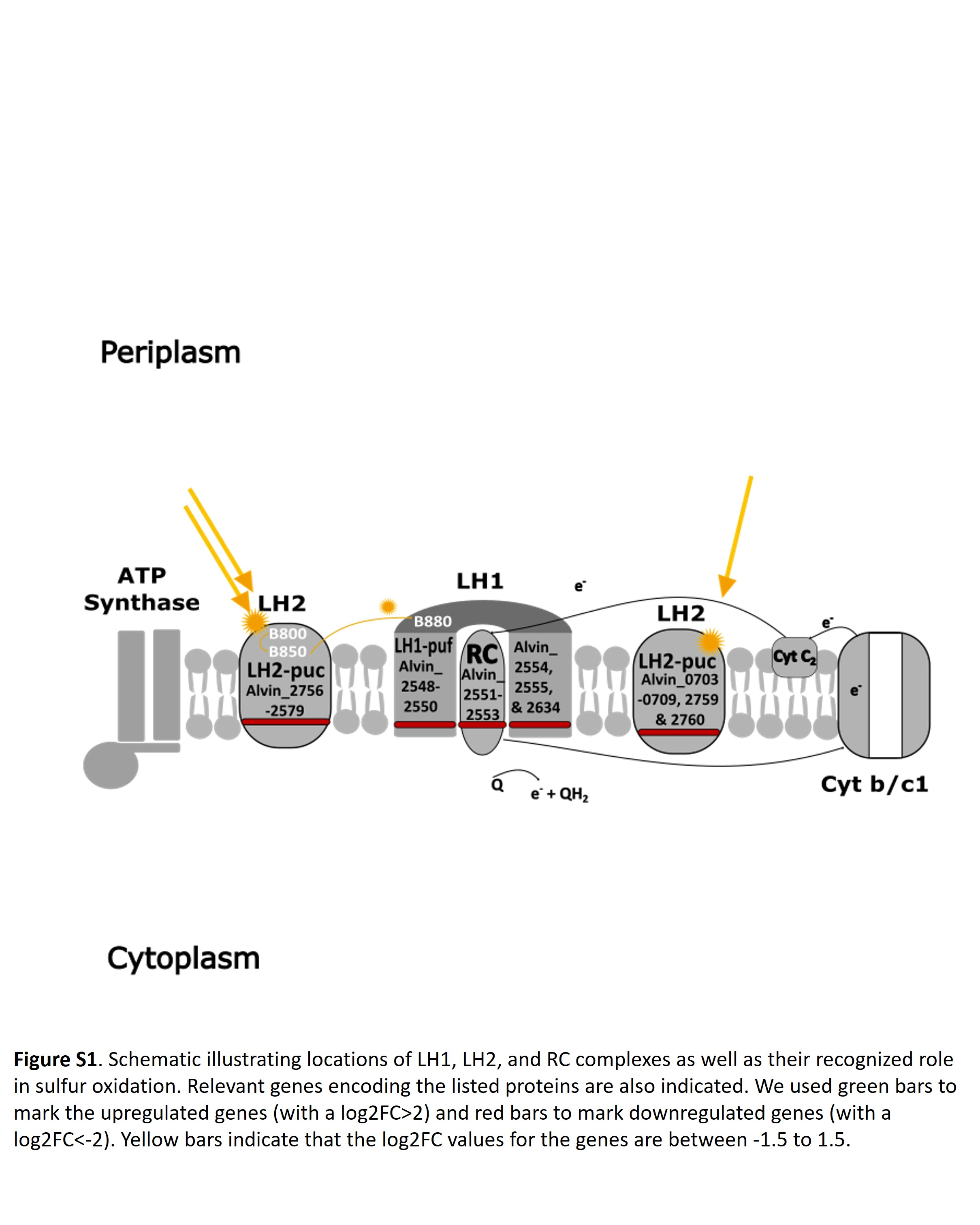

Supplement: Fig. S1 — Schematic illustrating locations of LH1, LH2, and RC complexes as well as their recognized role in sulfur oxidation. [file aem.00863-24-s0001.tif]

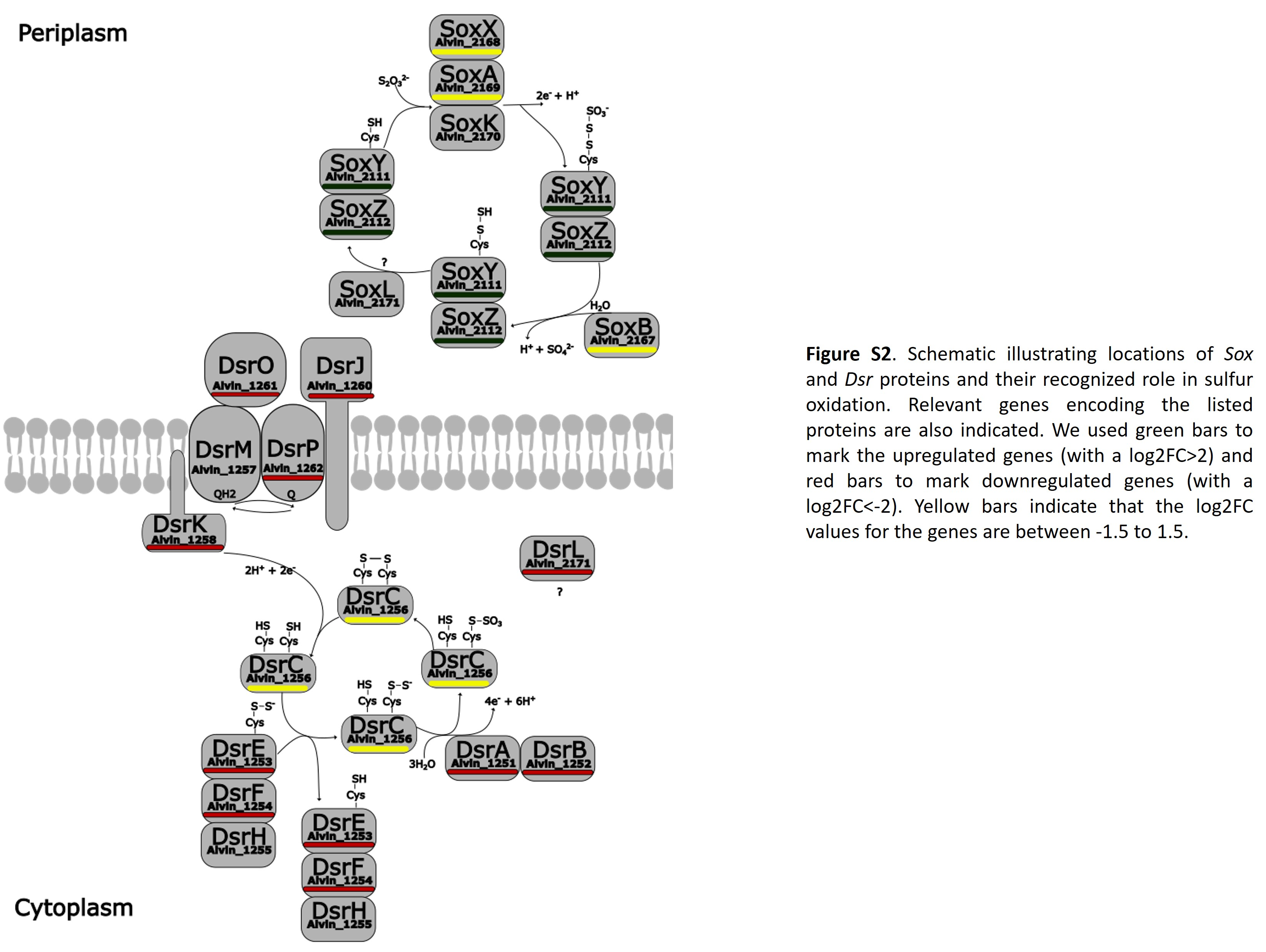

Supplement: Fig. S2 — Schematic illustrating locations of Sox and Dsr proteins and their recognized role in sulfur oxidation. [file aem.00863-24-s0002.tif]
