# Supplementary material for: Evidence for autotrophic growth of purple sulfur bacteria using pyrite as electron and sulfur source
Source: Appl Environ Microbiol. 2024 Jun 20;90(7):e00863-24. doi: 10.1128/aem.00863-24 (PMC11267869; doi:10.1128/aem.00863-24)

Supplemental Information

The ingredients of the mineral mix are listed below.

Chemical	Concentration (g/L)	Molecular weight
EDTA	0.2	292.24
$MgSO_4 \cdot 7H_2O$	3	246.4746
$MnSO_4 \cdot H_2O$	0.5	169.0159
NaCl	1	58.4428
$FeSO_4 \cdot 7H_2O$	0.1	278.01456
$Co(NO_3)_2 \cdot 6H_2O$	0.1	291.03
$CaCl_2$	0.1	110.98
$ZnSO_4 \cdot 7H_2O$	*0.1*	287.5496
$CuSO_4 \cdot 5H_2O$	0.01	249.72
$AlK(SO_4)_2$	0.01	258.205
H_3BO_3	0.01	61.83
$Na_2MoO_4 \cdot 2H_2O$	0.01	241.956
Na_2SeO_3	0.001	172.94
$Na_2WO_4 \cdot 2H_2O$	0.01	329.846
$NiCl_2 \cdot 6H_2O$	0.02	237.69

The pH was monitored for the control and pyrite cell culture media, which remained within a range of ~7.0-7.5.

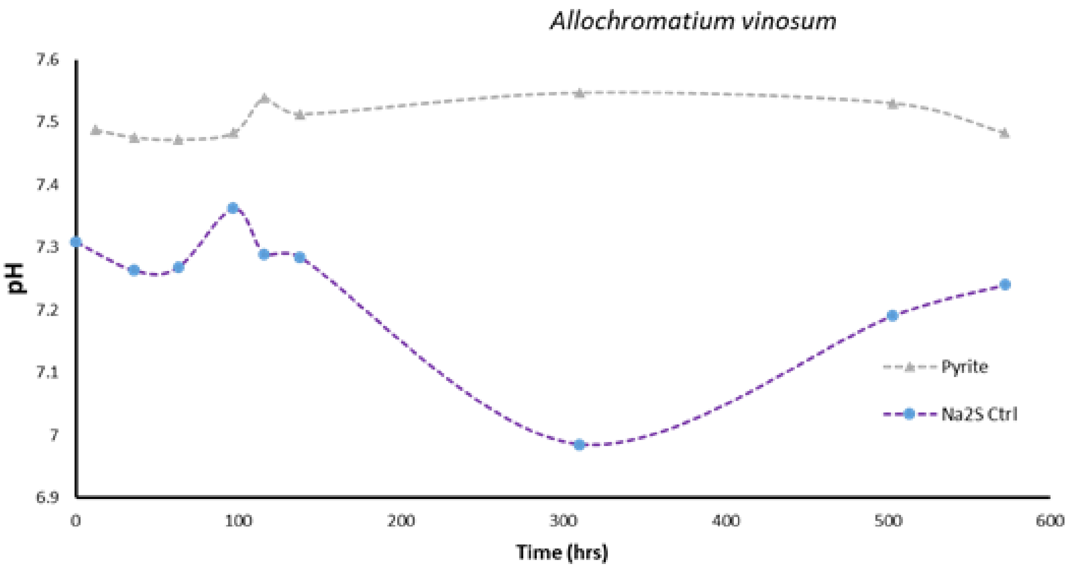

The light source used in the cell culture experiments were examined using a hyperspectral imaging system. The “no filter” data represents the wavelength distribution of the light source.

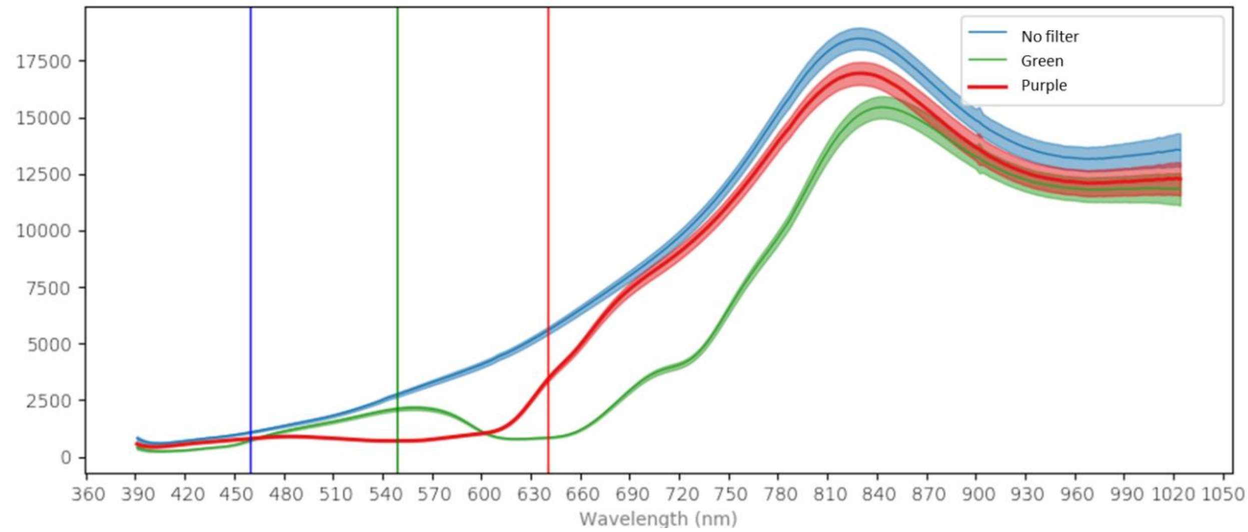

Supplement: Supplemental material — Ingredients of mineral mix, and graph of pH for control and pyrite cell culture media. [file aem.00863-24-s0003.pdf]
