# Supplementary material for: Evidence for autotrophic growth of purple sulfur bacteria using pyrite as electron and sulfur source
Source: Appl Environ Microbiol. 2024 Jun 20;90(7):e00863-24. doi: 10.1128/aem.00863-24 (PMC11267869; doi:10.1128/aem.00863-24)
Supplement: Table S3 — Compiled list of 43 putative c-type cytochromes in A. vinosum and their corresponding gene expression status in the pyrite culture samples. [file aem.00863-24-s0006.pdf]

Table S3. Compiled list of 43 putative *c*-type cytochromes in *A. vinosum* and their corresponding gene expression status in the pyrite culture samples. The number of CXXCH motifs indicate predicted number of hemes in the protein. Whether the cytochrome has lipid-binding motif (LXXC) and/or SignalP is also indicated. SignalP indicates detection of 1 of 5 signal peptides according to (Teufel *et al.* 2022). The values in the last column (bracketed) are fold changes in relevant gene expressions of the pyrite samples versus positive controls (i.e., the cells in positive controls used soluble sulfide as sulfur and electron donor).

Gene	Position	Description	Number of CXXCH motif	LXXC motif	NCBI Protein ID	SignalP	Upregulated in cells on pyrite?	upregulated in cells on elemental sulfur?
Alvin_0018	76..80	Di-heme cytochrome c peroxidase	2		ADC60990		Yes (9.4)	Yes (2.2)
	258..262							
Alvin_0020	56..60	Diheme cytochrome c	2	Yes	ADC60992	Yes	Yes (20.8)	No (0.8)
	165..169							
Alvin_0022	48..52	Domain of unknown function DUF1924	1		ADC60994	Yes	Yes (137.6)	No (0.08)
Alvin_0023	54..58	Diheme cytochrome c	2		ADC60995	Yes	Yes (46.5)	No (0.7)
	155..159							
Alvin_0070	50..54	cytochrome c1	1		ADC61042	Yes	Yes (1.5)	No (0.2)
Alvin_0071	9..13	Glutathione S-transferase domain protein	1	Yes	ADC61043		Yes (1.2)	No (0.3)
Alvin_0091	76..80	Thiosulfate dehydrogenase	2		ADC61061	Yes	Yes (2.6)	Yes (1.7)
	187..191							
Alvin_0350	159..163	nicotinate-nucleotide pyrophosphorylase	1		ADC61311		Yes (1.1)	N/A
Alvin_0439	43..47	transmembrane region and signal peptide prediction	1	Yes	ADC61398	Yes	No (0.8)	No (0.1)
Alvin_0679	76..80	Cytochrome-c peroxidase	2	Yes	ADC61628	Yes	No (0.9)	No (0.6)
	222..226							
Alvin_0782	105..109	cytochrome c oxidase, cbb3-type, subunit II	1		ADC61729		Yes (2.2)	Yes (6.2)
Alvin_0784	120..124	cytochrome c class I	2		ADC61731		Yes (1.8)	Yes (6.2)
	206..210							
Alvin_1073	877..881	FAD linked oxidase domain protein	1	Yes	ADC62012		No (0.9)	No (0.4)
Alvin_1093	36..40	cytochrome c class I	2		ADC62032	Yes	Yes (175.0)	Yes (4.5)
	126..130							

Alvin_1095	44..48	NapC/NirT cytochrome c domain protein	4	Yes	ADC62034		Yes (41.8)	Yes (9.6)
	73..77							
	133..137							
	165..169							
Alvin_1259	633..637	DsrL	1		ADC62198		No (0.2)	No (0.2)
Alvin_1260	83..87	DsrJ	3	Yes	ADC62199	Yes	No (0.2)	No (0.1)
	106..110							
	119..123							
Alvin_1395	72..76	cytochrome c family protein	8	Yes	ADC62330	Yes	No (0.5)	No (0.01)
	122..126							
	146..150							
	195..199							
	237..241							
	275..279							
	303..307							
	489..493							
Alvin_1402	172..176	Fe-S cluster assembly protein NifU	1	Yes	ADC62337		No (1.0)	Yes (1.3)
Alvin_1452	46..50	conserved hypothetical protein	7		ADC62387	Yes	Yes (1.7)	Yes (1.1)
	85..89							
	109..113							
	151..155							
	180..184							
	210..214							
	238..242							
Alvin_1454	130..134	hypothetical protein	8		ADC62389		Yes (2.6)	No (1.0)
	230..234							
	262..266							
	306..310							
	360..364							
	392..396							
	419..423							
	681..685							
Alvin_1467	37..41	Alcohol dehydrogenase GroES domain protein	2	Yes	ADC62401		Yes (2.4)	Yes (5.5)
	96..100							

Alvin_1573	150..154	methyl-accepting chemotaxis sensory transducer	1		ADC62506		Yes (1.6)	No (0.8)
Alvin_1694	24..28	cytochrome c class I	1		ADC62626	Yes	No (0.5)	Yes (3.0)
Alvin_1837	50..54	putative lipoprotein	2	Yes	ADC62762	Yes	Yes (1.6)	Yes (1.1)
	389..393							
Alvin_1846	57..61	cytochrome c class I	2		ADC62771	Yes	Yes (1.8)	Yes (1.5)
	151..155							
Alvin_1867	17..21	NADH ubiquinone oxidoreductase 20 kDa subunit	1	Yes	ADC62792		Yes (2.2)	N/A
Alvin_1971	87..91	Coproporphyrinogen dehydrogenase	1	Yes	ADC62895		Yes (2.6)	Yes (5.5)
Alvin_2064	61..65	protein of unknown function DUF255	1	Yes	ADC62986		Yes (1.7)	Yes (4.4)
Alvin_2168	56..60	SoxX	1	Yes	ADC63089	Yes	Yes (2.1)	Yes (5.7)
Alvin_2169	195..199	SoxA	1		ADC63090	Yes	Yes (2.3)	Yes (5.4)
Alvin_2172	89..93	HNH endonuclease	1		ADC63093		No (0.5)	Yes (1.1)
Alvin_2201	150..154	conserved hypothetical protein	1		ADC63122	Yes	No (0.6)	No (0.7)
Alvin_2458	41..45	NapC/NirT cytochrome c domain protein	4		ADC63370		Yes (1.9)	Yes (1.7)
	71..75							
	125..129							
	161..165							
Alvin_2459	46..50	hypothetical protein	1		ADC63371	Yes	Yes (1.8)	No (0.9)
Alvin_2490	13..17	4Fe-4S ferredoxin iron-sulfur binding domain protein	1	Yes	ADC63402		No (0.3)	No (0.2)
Alvin_2551	107..111	photosynthetic reaction center cytochrome c subunit	4	Yes	ADC63461	Yes	No (0.2)	No (0.005)
	152..156							
	247..251							
	307..311							
Alvin_2765	144..148	cytochrome c prime	1		ADC63674	Yes	Yes (2.4)	Yes (2.6)
Alvin_2879	42..46	cytochrome c class I	2		ADC63784	Yes	No (1.0)	No (0.4)
	138..142							
Alvin_3050	37..41	ribosomal protein L31	1		ADC63950		No (0.4)	No (0.7)
Alvin_3069	41..45	Thioredoxin domain protein	1		ADC63969		Yes (1.2)	Yes (6.6)
Alvin_3120	550..554	type II secretion system protein E	1	Yes	ADC64019		No (0.8)	No (0.7)

Alvin_3135	93..97	Radical SAM domain protein	1	Yes	ADC64033		Yes (1.6)	N/A
